# FoxO1-mediated autophagy is required for NK cell development and innate immunity

**DOI:** 10.1038/ncomms11023

**Published:** 2016-03-24

**Authors:** Shuo Wang, Pengyan Xia, Guanling Huang, Pingping Zhu, Jing Liu, Buqing Ye, Ying Du, Zusen Fan

**Affiliations:** 1 CAS Key Laboratory of Infection and Immunity, Institute of Biophysics, Chinese Academy of Sciences, Beijing 100101, China

## Abstract

Natural killer (NK) cells exert a crucial role in early immune responses as a major innate effector component. However, the underlying mechanisms of NK cell development remain largely elusive. Here we show that robust autophagy appears in the stage of immature NK cells (iNKs), which is required for NK cell development. Autophagy defects result in damaged mitochondria and accumulation of reactive oxygen species (ROS) that leads to apoptosis of NK cells. Autophagy protects NK cell viability during development through removal of damaged mitochondria and intracellular ROS. Phosphorylated Forkhead box O (FoxO)1 is located to the cytoplasm of iNKs and interacts with Atg7, leading to induction of autophagy. FoxO1 deficiency or an inactive FoxO1^AAA^ mutant abrogates autophagy initiation in iNKs and impairs NK cell development and viral clearance. Therefore we conclude that FoxO1-mediated autophagy is required for NK cell development and NK cell-induced innate immunity.

## Abstract (web summary)

Natural killer cells are a major component of the innate immune response. Here, Wang *et al*. show that natural killer cell development requires robust activation of autophagy at the immature stage where it acts to remove damaged mitochondria and reduces levels of reactive oxygen species.

## Graphical abstract

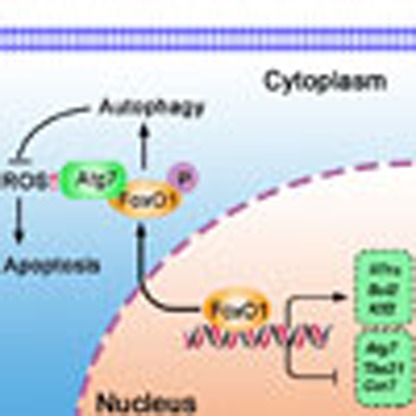

Natural killer (NK) cells, a major component of innate immunity, serve as the first line of defence against transformed tumours and virus-infected cells^1^,^2^. NK cells were recently defined as a part of the group 1 innate lymphoid cells according to their cytokine secretion pattern^3^. Cytokine secretion and granule-mediated cytotoxicity are the two major effector functions of NK cells, which are critical for early immune responses^2^,^4^. NK cells also play a pivotal role in orchestrating adaptive immunity^5^,^6^. Recent studies reported that NK cells obtain antigen specificity and develop into long-lived memory cells under antigen stimulation, displaying their adaptive features of NK cells^6^. Like leukocyte populations, NK cells derive from hematopoietic stem cells (HSCs) in the bone marrow (BM). Each step of NK cell development is finely regulated via signalling by various cytokines and transcription factors. Common lymphoid progenitors (CLPs) derived from multipotent progenitors can differentiate into NK progenitors (NKPs). NKPs express IL-15 receptor β chain (CD122) that allows them to respond to IL-15 (ref. 7). Under IL-15 signalling, NKPs thereafter develop into immature NK (iNKs) and mature NK cells (mNKs)^8^. In addition, transcription factors are needed for NK cell specification. Id2 and Ets1, for example, promote NK cell lineage commitment^7^. E4bp4 directs iNK to mNK transition^9^,^10^. Gata-3, Eomes, T-bet and TOX are required for the maturation of NK cells^11^,^12^,^13^,^14^. However, it is unclear whether Forkhead box O (FoxO) family transcription factors play a role in NK cell development.

Mammalian FoxO transcription factors, containing FoxO1, FoxO3, FoxO4 and FoxO6, are homologues of the *Caenorhabditis elegans* FoxO ortholog Daf16 that is crucial for Dauer larval stage formation^15^. The Dauer larva, an alternative developmental stage of nematode worms, arrests development and allows survival in harsh conditions. Most FoxO members harbour an evolutionally conserved role in the modulation of nutrient sensing and stress responses. For instance, FoxO1 plays a critical role in cell cycle arrest, oxidative stress resistance and regulation of metabolism^16^. *FoxO1* knockout mice exhibit vascular defects and die at E10.5 (ref. 16). Moreover, FoxO1 is essential for the regulation of homing and survival of naive T cells^15^. FoxO1 deficiency in Treg cells can switch their inhibitory functions to effector functions^17^. In addition, FoxO1 also regulates memory CD8^+^ T-cell responses^18^. FoxO1 is also indispensable for early B-cell development and its peripheral functions^19^. Except for the transcriptional activity of FoxO1, cytosolic FoxO1 is able to induce autophagy in human cancer cells upon oxidative stress or serum starvation^20^. Additionally, several previous studies reported that another FoxO family member, FoxO3, is also involved in the autophagy induction in muscle cells^21^. A recent study showed that FoxO3a triggers autophagy that is essential for the life-long maintenance of HSCs^22^.

Macroautophagy (hereafter referred to as autophagy) is an evolutionarily conserved process that degrades long-lived proteins and unwanted organelles to recycle cellular components for survival and homoeostasis^23^. Autophagy participates in a variety of physiological processes, including lymphocyte development, embryonic development, cell reprogramming, tumour suppression and microbial clearance^24^,^25^. Moreover, autophagy is required for the maintenance of HSCs, T and B cells^23^. Recently, autophagy was shown to be required for plasma cell homoeostasis and humoral immunity^26^. During autophagy, autophagy-related genes, including Atg7, Atg5 and Atg3, are required for autophagosome formation^27^. However, whether autophagy is involved in NK cell development and effector functions are still unknown.

Here we show that robust autophagy appears in iNKs and is required for NK cell development. Phosphorylated FoxO1 is located to the cytoplasm of iNKs and interacts with Atg7, which promotes autophagy induction. FoxO1 deficiency or an inactive FoxO1^AAA^ mutant abolishes autophagy initiation in iNKs and impairs NK cell development and viral clearance.

## Results

### Robust autophagy appears in iNKs

**Figure 1:**
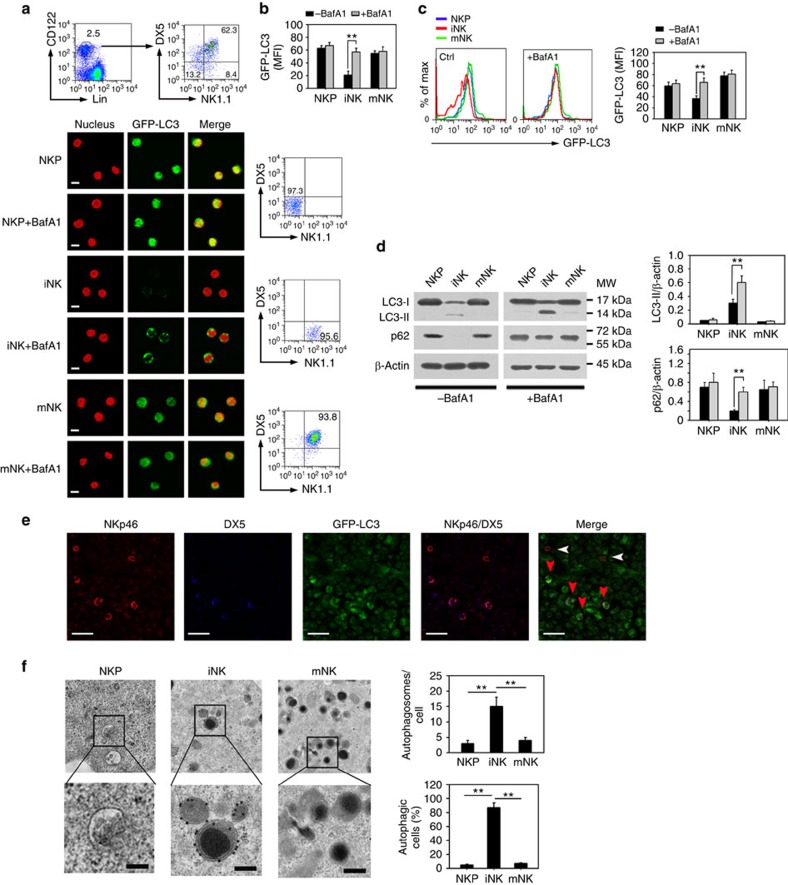
Robust autophagy occurs in the stage of iNK cells. (**a**,**b**) Total BM cells from GFP-LC3 transgenic mice were flushed from mouse femurs and stained with antibodies against lineage, CD122, NK1.1 and DX5. Different stages of NK cells were isolated by flow cytometry. Purity of each NK population was confirmed by flow cytometry analysis. Cells were fixed or cultured with 20 nM Bafilomycin A1 (BafA1) for 4 h and visualized by confocal microscopy. NKP, Lin^−^CD122^+^NK1.1^−^DX5^−^; iNK, Lin^−^CD122^+^NK1.1^+^DX5^−^; mNK, Lin^−^CD122^+^NK1.1^+^DX5^+^. Lineage cocktails (Lin) included CD3 (17A2), CD19 (1D3), TER-119 (TER-119), and Gr-1 (RB6-8C5). Nuclei were stained with propidium iodide (PI). Scale bar, 5 μm. Medians of fluorescence intensity (MFI) of each cell were calculated and shown as means±s.d. **P<0.01 (**b**). (**c**) NK cells from GFP-LC3 mice were treated as above, MFI was analysed by flow cytometry and shown as means±s.d. **P<0.01. (**d**) NK cells (1 × 10^5^) at three stages were isolated from wild-type (WT) mice and cultured with or without BafA1 for 4 h followed by immunoblotting. Bands were quantified by Image J and shown as means±s.d. **P<0.01. (**e**) BM sections from femurs of GFP-LC3 mice were stained with antibodies against NKp46, DX5, and GFP. Scale bar, 20 μm. White arrow head denotes iNK cells (NKp46^+^DX5^−^); Red arrow head indicates mNK cells (NKp46^+^DX5^+^). (**f**) NK cells of the indicated stages were stained with anti-LC3 antibody and visualized by immuno-electron microscopy. Enlarged fields showed cytosolic organelles (endosomes in NKP, autophagosomes in iNKs and granules in mNKs). Scale bar, 200 nm. Number of autophagosomes per cell from more than 50 cells of different NK stages was calculated and shown as means±s.d. The percentage of autophagic cells in the indicated stages was shown as means±s.d. (*n*>100). **P<0.01. Data were repeated for three times with similar results. For **c** and **d**, a two-tailed unpaired Student's *t*-test was used; for **f**, a two-way analysis of variance *post hoc* Bonferroni test was used.

To examine whether autophagy occurs during NK cell development, we tested for autophagic activity in different stages of NK cells by using green fluorescent protein (GFP)-fused LC3 (GFP-LC3) transgenic mice, which have been used to monitor autophagic activity. Turnover of GFP-LC3 fluorescence represents robust autophagic flux^28^. We found that the GFP fluorescence signal was remarkably reduced in iNKs (Fig. 1a–c), whereas the GFP signal was apparent in NKPs and mNKs. Four independent experiments obtained similar observations. Bafilomycin A1 (BafA1) serves as an autolysosome inhibitor^28^. With BafA1 treatment, autophagic puncta significantly occurred in iNK cells, but not in NKPs and mNKs (Fig. 1a–c), indicating that autophagic flux is increased in iNKs. These data suggest that robust autophagy occurs in the stage of iNKs.

LC3 conversion and p62 degradation are typical indicators of autophagy^29^. Consistently, we observed that obvious conversion of LC3-I to LC3-II appeared in iNKs (Fig. 1d), and p62, a substrate of autophagosomes, was almost hydrolysed in these cells. Upon BafA1 treatment, the degradation of LC3-II and p62 were abrogated in iNKs, indicating enhanced autophagic flux in these cells. By contrast, conversion of LC3 and degradation of p62 did not appear in NKPs and mNKs. Additionally, the mRNA levels of LC3 and p62 did not apparently change in iNKs (Supplementary Fig. 1a). We noticed that NKp46 distributed in most iNKs and all mature NK cells, but not in NKPs (Supplementary Fig. 1b), consistent with a previous report^30^. Since an anti-NK1.1 antibody did not work for immunohistochemistry, we then used an anti-NKp46 antibody in place of anti-NK1.1 antibody to detect iNK and mature NK cell populations by immunohistochemistry. Consequently, we observed that iNKs displayed weak GFP-LC3 signals in BM sections (Fig. 1e), whereas mNKs showed strong GFP-LC3 fluorescence. We further analysed NK cell ultrastructures via immuno-eletron microscopy using anti-LC3 antibody. As expected, substantial LC3-positive autophagosome structures occurred in iNKs (Fig. 1f), and over 80% of iNKs displayed greater numbers of autophagosomes. By contrast, there were no overt LC3-positive autophagosome structures in NKPs or mNKs. Taken together, these results suggest that iNKs undergo robust autophagy during NK cell development.

### Autophagy is required for NK cell development

**Figure 2:**
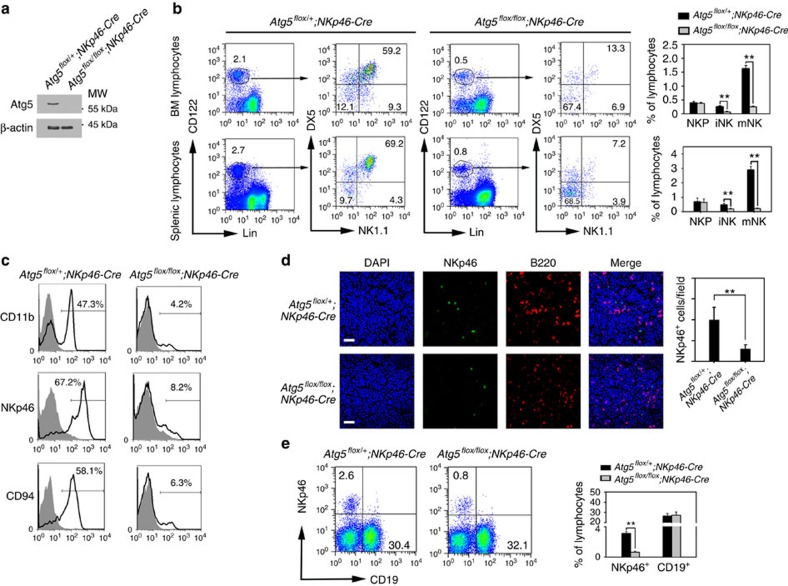
Autophagy is essential for NK cell development. (**a**) Atg5 in the CD3^−^NK1.1^+^ NK cells from the BM of *Atg5*^*flox/+*^*;NKp46-Cre* and *Atg5*^*flox/flox*^*;NKp46-Cre* mice was probed by western blotting. β-Actin was used as a loading control. (**b**) Defect of NK cell development in *Atg5*-deficient NK cells. Three stages of NK cells in *Atg5*^*flox/+*^*;NKp46-Cre* mice (*n*=10) and *Atg5*^*flox/flox*^*;NKp46-Cre* mice (*n*=10) were gated from propidium iodide (PI)- negative BM and splenic lymphocytes and analysed by flow cytometry. Lin=CD3 (17A2), CD19 (1D3), TER-119 (TER-119), Gr-1 (RB6-8C5). (**c**) Mature NK markers are abolished in Atg5-deficient NK cells. Lin^−^CD122^+^ gated BM lymphocytes from *Atg5*^*flox/+*^*;NKp46-Cre* mice and *Atg5*^*flox/flox*^*;NKp46-Cre* mice were analysed with NK cell markers by flow cytometry. Grey histograms depict isotype control of each antibody. (**d**) NK or B cells in BM sections were stained with anti-NKp46 and anti-B220 antibodies. Nuclei were stained with DAPI. Scale bar, 30 μm. More than 50 fields were counted from 5 mice for each mouse strain. (**e**) NK (NKp46^+^) and B (CD19^+^) cells in *Atg5*^*flox/+*^*;NKp46-Cre* and *Atg5*^*flox/flox*^*;NKp46-Cre* mice were analysed by flow cytometry. Percentage of NKp46^+^ cells among lymphocytes was calculated and shown as means±s.d. **P<0.01. For **b**,**d** and **e**, a two-tailed unpaired Student's *t*-test was used.

To determine the function of autophagy in iNKs, we generated mice that lacked autophagic activity in iNKs by crossing *Atg5*^*flox/flox*^ mice with mice expressing Cre recombinase driven by the NK cell-specific NKp46 promoter (*NKp46-Cre*)^30^. Given that the NKp46 receptor is expressed in the stages of iNKs and mNKs, Atg5 was successfully deleted in iNKs and mNKs in *Atg5*^*flox/flox*^*;NKp46-Cre* mice (Fig. 2a; Supplementary Fig. 2a). We observed that littermate *Atg5*^*flox/+*^*;NKp46-Cre* mice behaved similar to WT *Atg5*^*+/+*^ mice. We therefore used *Atg5*^*flox/+*^*;NKp46-Cre* mice as littermate controls. Notably, total lymphocyte numbers in *Atg5*^*flox/flox*^*;NKp46-Cre* mice were comparable to those of *Atg5*^*flox/+*^*;NKp46-Cre* control mice. Interestingly, we observed that *Atg5*^*flox/flox*^*;NKp46-Cre* mice had dramatically lowered percentages of iNKs and mNKs both in the BM and spleen (Fig. 2b), indicating reduced numbers of iNKs and mNKs accordingly. By contrast, Cre expression alone did not impact NK cell development and restrictive expression of NKp46 (Supplementary Fig. 2b,c). Moreover, several surface markers of iNKs and mNKs in *Atg5*^*flox/flox*^*;NKp46-Cre* mice were remarkably reduced by flow cytometry analysis (Fig. 2c), suggesting that *Atg5* deficiency impaired the mature transition from NKPs to the stages of iNKs and mNKs.

We next stained NK and B cells in BM sections. Expectedly, the number of NKp46^+^ cells was dramatically reduced in *Atg5*^*flox/flox*^*;NKp46-Cre* mice compared with *Atg5*^*flox/+*^*;NKp46-Cre* mice (Fig. 2d), whereas we did not detect a difference in the number of B cells. These results were further confirmed by flow cytometry analysis (Fig. 2e). The frequencies of other cell types, including HSCs, CLPs, myeloid cells in the BM were not significantly different in *Atg5*^*flox/flox*^*;NKp46-Cre* mice (Supplementary Fig. 3a). Atg3 and Atg7 are involved in autophagosome formation^31^. Expectedly, silencing of Atg3- or Atg7-impaired NK cell development (Supplementary Fig. 3b,c). These data indicate that autophagy is required for NK cell development.

**Figure 3:**
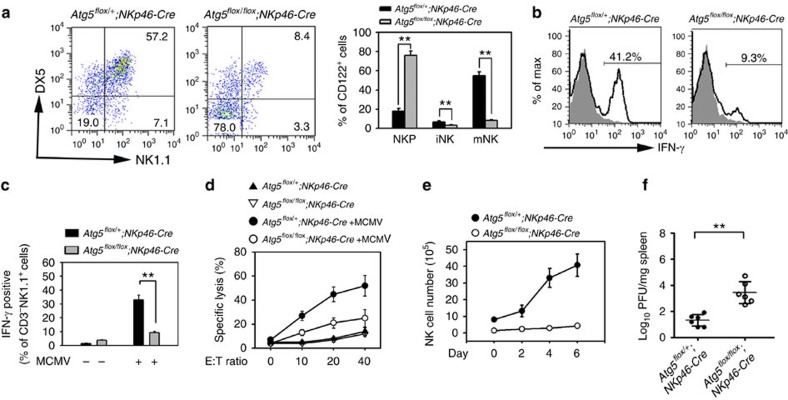
Autophagy is required for NK cell effector functions. (**a**) NKP cells from *Atg5*^*flox/+*^*;NKp46-Cre* and *Atg5*^*flox/flox*^*;NKp46-Cre* mice were cultured in medium containing 10 ng ml^−1^ IL-15 on OP9 stroma cells for 7 days followed by cytometry. (**b**) CD3^−^NK1.1^+^ NK cells were isolated from *Atg5*^*flox/+*^*;NKp46-Cre* and *Atg5*^*flox/flox*^*;NKp46-Cre* mice and cultured in medium containing 10 ng ml^−1^ IL-12 and 10 ng ml^−1^ IL-18 for 12 h followed by brefeldin A (BFA) treatment, and stained for intracellular IFN-γ. Grey histograms depict isotype control of anti-IFN-γ antibody. (**c**) MCMV-induced IFN-γ is impaired in *Atg5*^*flox/flox*^*;NKp46-Cre* mice. Mice were infected with 1 × 10^5^ PFU MCMV for 3 days, and intracellular IFN-γ of NK1.1^+^ cells were tested by flow cytometry. Data were repeated for three times with similar results, and shown as means±s.d. **P<0.01. (**d**) *Atg5*^*flox/+*^*;NKp46-Cre* and *Atg5*^*flox/flox*^*;NKp46-Cre* mice were infected with MCMV for 3 days. NK cells were separated and incubated with ^51^Cr-labelled Yac1 cells with indicated E:T ratios. Specific lysis of Yac1 was analysed by ^51^Cr release, and shown as means±s.d. (**e**) *Atg5*^*flox/+*^*;NKp46-Cre* and *Atg5*^*flox/flox*^*;NKp46-Cre* mice were infected with MCMV for the indicated days, and NK cell numbers were analysed by flow cytometry. (**f**) Virus titers from MCMV-infected *Atg5*^*flox/+*^*;NKp46-Cre* mice and *Atg5*^*flox/flox*^*;NKp46-Cre* mice were analysed after three days' infection. *n*=6 mice for each group. **P<0.01. All data are representative of at least three independent experiments and calculated data are shown as means±s.d. **P<0.01. For **a**,**c** and **f**, a two-tailed unpaired Student's *t*-test was used.

Under stimulation of IL-15 *in vitro*, NKPs can develop to subsequent stages of iNKs and mNKs^32^. We isolated NKPs from *Atg5*^*flox/flox*^*;NKp46-Cre* mouse BM and cultured them with IL-15 *in vitro*. *Atg5*^*flox/flox*^*;NKp46-Cre* NKPs failed to generate iNKs and mNKs (Fig. 3a), while *Atg5*^*flox/+*^*;NKp46-Cre* NKPs were able to induce formation of iNKs and mNKs. In addition, we found that *Atg5*^*flox/flox*^*;NKp46-Cre* NKs abolished IFN-γ production (Fig. 3b), whereas *Atg5*^*flox/+*^*;NKp46-Cre* NKs produced a large amount of IFN-γ. Murine cytomegalovirus (MCMV) infection induces production of pro-inflammatory cytokines at an early phase that in turn stimulate NK cells to produce other antiviral cytokines for NK cell expansion and upregulation expression of activating receptors^33^. Thereby NK cells play a critical role in the early control of MCMV infection. We found that *Atg5*^*flox/flox*^*;NKp46-Cre* NK cells produced much less IFN-γ upon MCMV stimulation compared with control *Atg5*^*flox/+*^*;NKp46-Cre* NKs (Fig. 3c). The cytotoxicity and expansion of *Atg5*^*flox/flox*^*;NKp46-Cre* NK cells were severely impaired (Fig. 3d,e). Importantly, *Atg5*^*flox/flox*^*;NKp46-Cre* mice were more susceptible to MCMV infection (Fig. 3f). Finally, we further validated that autophagy was required for NK effector functions. We isolated NK cells from *Atg5*^*flox/flox*^*;NKp46-Cre* mice and *Atg5*^*flox/+*^*;NKp46-Cre* mice and treated with IL-12 and IL-18 for 12 h. Atg5-deficient mNKs displayed impaired IFN-γ secretion and cytotoxicity (Supplementary Fig. 4a,b). Overall, autophagic activity is required for NK cell development and its effector functions.

### Autophagy protects NK cells against ROS-induced apoptosis

**Figure 4:**
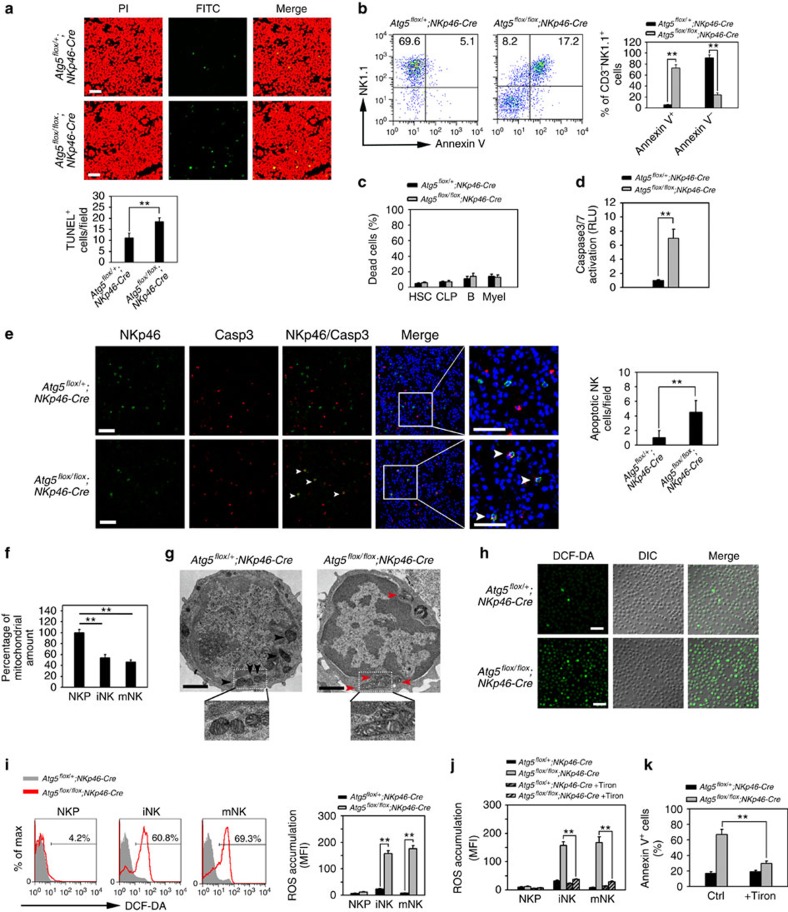
Autophagy protects NK cells from apoptosis during development. (**a**) BM sections of the indicated mice were subjected to TUNEL assay. Number of TUNEL positive cells per field was calculated as means±s.d. **P<0.01. Scale bar, 30 μm. (**b**) NK cells (Lin^−^CD122^+^) in *Atg5*^*flox/+*^*;NKp46-Cre* mice (*n*=6) and *Atg5*^*flox/flox*^*;NKp46-Cre* mice (*n*=6) were stained with anti-NK1.1 and AnnexinV. Calculated data are shown as means±s.d. **P<0.01. (**c**) HSC, CLP, B cells and myeloid cells (Myel) from the indicated mice (*n*=6) were stained with AnnexinV as above. (**d**) NK (CD3^−^NK1.1^+^) cells in the indicated mice were isolated for caspase activity assay. (**e**) BM sections from the indicated mice were stained with anti-NKp46 and anti-activated caspase3. Scale bare, 30 μm. White arrow head indicates apoptotic NK cells. Apoptotic NK cells were calculated and shown as means±s.d. **P<0.01. (**f**) NK cells at different stages were isolated from BM of WT mice and stained with MitoTracker Red for flow cytometry. (**g**) Mitochondria in iNK cells were visualized by electron microscopy. Enlarged fields showed mitochondria in cytosol. Black arrow head indicates normal mitochondria. Red arrow head denotes damaged mitochondria. Scale bar, 1 μm. (**h**) NK (CD3^−^NK1.1^+^) cells were isolated from the BM of indicated mice followed by DCF-DA staining. Scale bar, 20 μm. (**i**) ROS levels of NK cells at indicated stages were analysed by DCF-DA staining. MFI was calculated and shown as means±s.d. **P<0.01. (**j**) NKP cells were cultured with or without 10 mM Tiron for *in vitro* development assay for ROS assay. (**k**) NKP cells were cultured with 10 ng ml^−1^ IL-15 in the present or absence of 10 nM Tiron followed by apoptosis assay for CD3^−^NK1.1^+^ cells. All data are representative of at least three independent experiments and calculated data are shown as means±s.d. **P<0.01. For **a** and **e**, More than 50 fields from five slides for each mouse strain were counted. For **b**–**e** and **i**–**k**, a two-tailed unpaired Student's *t*-test was used; for **f**, a two-way analysis of variance *post hoc* Bonferroni test was used.

We next wanted to test the physiological role of autophagy in the progression of NK cell development. We noticed that many more apoptotic cells appeared in the BM of *Atg5*^*flox/flox*^*;NKp46-Cre* mice compared with *Atg5*^*flox/+*^*;NKp46-Cre* control mice (Fig. 4a). Indeed, the number of AnnexinV-positive NK1.1^+^ NK cells was dramatically increased in *Atg5*^*flox/flox*^*;NKp46-Cre* mice according to flow cytometry analysis (Fig. 4b). However, the other cell types we detected in the BM of *Atg5*^*flox/flox*^*;NKp46-Cre* mice did not exhibit overt dead cells (Fig. 4c). Additionally, *Atg5*^*flox/flox*^*;NKp46-Cre* NK cells displayed high activation of caspase 3 and caspase 7 (Fig. 4d), whereas *Atg5*^*flox/+*^*;NKp46-Cre* NKs had basal levels. To further verify that NK cells underwent apoptosis in BM, we analysed apoptotic features of NK cells in BM sections. Consistently, most of NKp46^+^ NK cells in *Atg5*^*flox/flox*^*;NKp46-Cre* mice exhibited high caspase 3 activation compared with those of *Atg5*^*flox/+*^*;NKp46-Cre* mice (Fig. 4e). These results indicate that defective autophagy results in apoptosis of NK cells.

Autophagy contributes to clearance of damaged mitochondria and reactive oxygen species (ROS) during cell renovation^34^. Lack of autophagy causes damaged mitochondria and accumulation of ROS that leads to cell death. We observed that numbers of mitochondria were significantly reduced in iNKs and mNKs during NK cell maturation in *Atg5*^*flox/+*^*;NKp46-Cre* mice (Fig. 4f). However, *Atg5*-deficient iNK cells displayed numerous damaged mitochondria (Fig. 4g), whereas *Atg5*^*flox/+*^*;NKp46-Cre* iNK cells exhibited normal mitochondria. These data indicate a failure to clear damaged mitochondria in *Atg5*^*flox/flox*^*;NKp46-Cre* iNK cells. We then examined ROS levels at different stages of NK cell development in *Atg5*^*flox/flox*^*;NKp46-Cre* mice and *Atg5*^*flox/+*^*;NKp46-Cre* mice. As expected, ROS levels were much higher in *Atg5*^*flox/flox*^*;NKp46-Cre* iNKs and mNKs (Fig. 4h,i), whereas NKPs had no obvious ROS accumulation. Additionally, the ROS scavenger Tiron could eradicate ROS accumulation in *Atg5*^*flox/flox*^*;NKp46-Cre* NKs (Fig. 4j). Consequently, Tiron treatment dramatically reduced apoptosis of *Atg5*^*flox/flox*^*;NKp46-Cre* NKs (Fig. 4k), suggesting that ROS was the cause of apoptosis in *Atg5*^*flox/flox*^*;NKp46-Cre* NK cells. Taken together, these results suggest that autophagy is essential for clearance of damaged mitochondria and ROS of iNK cells during NK cell development.

### NK cells undergo mTOR-independent autophagy

**Figure 5:**
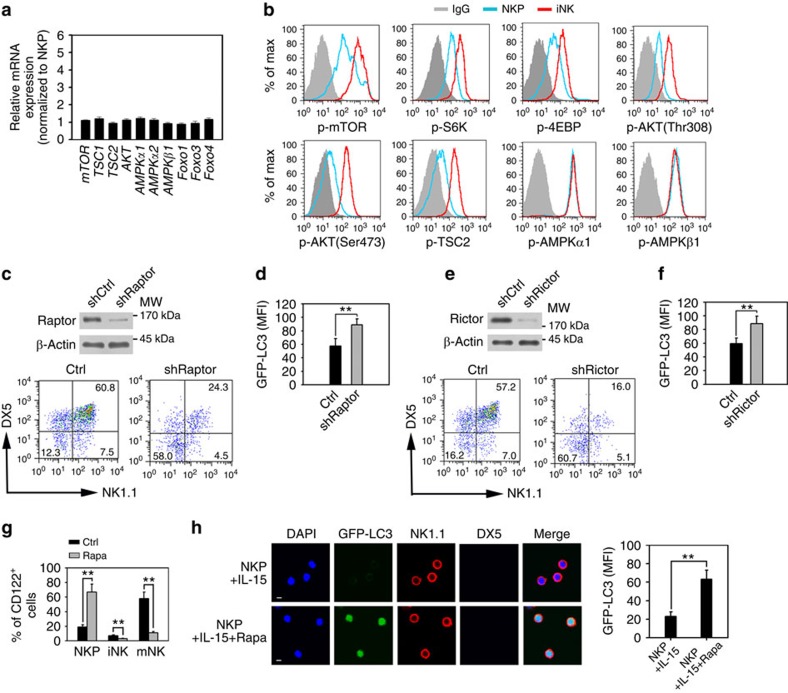
NK cells undergo mTOR-independent autophagy during development. (**a**) mRNA was extracted from isolated NKP and iNK cells followed by real-time PCR (RT-PCR) analysis. Relative mRNA expression of iNKs was normalized to that of NKPs. (**b**) NKP and iNK cells of WT mice were stained for the indicated antibodies and analysed by flow cytometry. (**c**) Raptor silencing inhibits NK cell development. NKP cells were infected with lentivirus encoding shRaptor and cultured with 10 ng ml^−1^ IL-15 for 7 days and analysed by flow cytometry. (**d**) Raptor depleted NKP cells from GFP-LC3 mice were subjected to *in vitro* development assay. GFP-LC3 MFI of iNK cells were analysed by flow cytometry. (**e**,**f**) Rictor-silenced NKP cells were infected with lentivirus encoding shRictor and treated as Raptor depleted NKP cells. (**g**) Inhibition of mTORC1 impairs NK cell development. NKP cells from WT mice were cultured with or without 1 μg ml^−1^ rapamycin (Rapa) for *in vitro* development assay. (**h**) NKP cells from GFP-LC3 mice were cultured with or without 1 μg ml^−1^ rapamycin for an *in vitro* development assay. GFP levels of iNK (NK1.1^+^DX5^−^) cells were analysed by confocal microscopy. GFP-LC3 MFI of iNKs was calculated and shown as means±s.d. **P<0.01. Scale bar, 5 μm. All data represent at least three independent experiments and calculated data are shown as means±s.d. **P<0.01. For **a**,**d** and **f**–**h**, a two-tailed unpaired Student's *t*-test was used.

To explore the underlying pathway of autophagy in iNKs, we next analysed expression profiles of autophagy-related genes. We observed no significant change in the expression of the autophagy-related genes we checked in iNKs (Fig. 5a). We further detected the activation levels of these autophagy-related proteins by analysing their phosphorylation states. Inhibition of mammalian target of rapamycin (mTOR) activation can initiate autophagy. Intriguingly, mTOR was highly phosphorylated in iNKs compared with NKPs (Fig. 5b). S6K, 4EBP and AKT are downstream substrates for mTOR^35^. Consequently, these mTOR substrates were also highly phosphorylated in iNKs (Fig. 5b). TSC2 is a downstream substrate of AKT (ref. 36). We found that TSC2 was also active in iNKs. However, the activation of AMPKα1 and AMPKβ1 was unchanged in iNKs compared with NKPs (Fig. 5b). These results suggest that the mTOR pathway is activated in iNKs.

mTOR exists in two distinct complexes, mTORC1 and mTORC2. mTORC1 phosphorylates S6K at Thr389 and mTORC2 phosphorylates AKT at Ser473, which consequently initiate the phosphorylation of AKT at Thr308. Inhibition of mTORC1 can initiate autophagy. While mTORC2 is able to modulate mTORC1 via AKT phosphorylation^35^. Raptor is a major component of mTORC1, while Rictor is a main component of mTORC2. We next wanted to test whether mTOR was involved in the regulation of NK cell autophagy or development. We observed that mTORC1 (Raptor) and mTORC2 (Rictor) were highly expressed in NKPs (Supplementary Fig. 4c). We knocked down Raptor or Rictor in NKPs, respectively. Silencing of Raptor abrogated NK cell development (Fig. 5c). Moreover, GFP-LC3 signals remained at a high level in Raptor-silenced NK cells (Fig. 5d), suggesting that Raptor depletion failed to induce autophagy in NK cells. Similar results were obtained in Rictor-silenced NKPs (Fig. 5e,f). These data indicate that the mTOR complexes are not implicated in the autophagy initiation, but they are involved in the progression of NK cell development.

Rapamycin has been widely used as an inhibitor of mTOR^37^. We added rapamycin to NKP culture media for an *in vitro* development assay. We observed that rapamycin treatment abolished NK cell development (Fig. 5g), rather than promoted apparent autophagy in iNKs (Fig. 5h). Altogether, these results suggest that induction of autophagy required for NK cell development occurs independently of mTOR.

### FoxO1 is highly phosphorylated in the cytoplasm of iNKs

**Figure 6:**
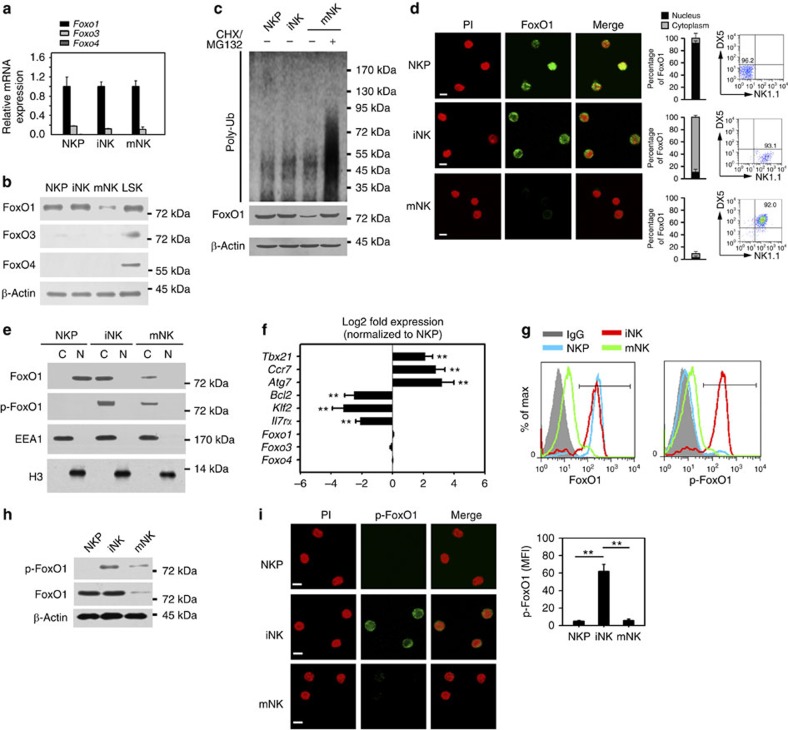
FoxO1 is highly expressed in the cytoplasm of iNKs. (**a**) mRNA was extracted from the indicated stages of NK cells followed by RT-PCR assay. Relative mRNA expression of NKs was normalized to that of CLPs Data are representative of three independent experiments and shown as means±s.d. (**b**) NK cells were subjected to western blotting. Hematopoietic progenitor cells (Lin^−^Sca1^+^cKit^+^ (LSK)) served as a positive control. (**c**) The indicated NK populations were isolated and mNKs were treated with 20 μg ml^−1^ CHX and 10 μm MG132 for 4 h followed by western blotting. (**d**) NK cells were isolated for immunostaining of FoxO1. Percentage of nuclear and cytoplasmic FoxO1 in cells was analysed. For a single cell, fluorescence intensities of nuclear FoxO1 (PI/FoxO1 positive areas, yellow) and cytosolic FoxO1 (FoxO1 positive areas, green) were analysed by Image J. Ratios of fluorescence intensities from nuclear or cytosolic FoxO1 were calculated from more than 50 cells for each NK cell population and shown as means±s.d. Scale bar, 5 μm. Purity of each NK population was confirmed by flow cytometry assay. (**e**) Nuclei and cytoplasm of different NK cells were separated followed by Western blotting. Cytoplasmic protein EEA1 and nuclear protein histone H3 served as fraction controls. C, cytoplasm; N, nucleus. (**f**) mRNA levels of the indicated genes during NKP to iNK transition was analysed by RT-PCR assay. Data were calculated as means±s.d. **P<0.01. (**g**,**h**) Different stages of NK cells were isolated for cytometry analysis (**g**) or immunoblotting (**h**). (**i**) FoxO1 is phosphorylated in the cytoplasm of iNKs. Different NK cells were separated for immunostaining. MFI of phosphorylated FoxO1 (p-FoxO1) was analysed and shown as means±s.d. **P<0.01. Scale bar, 5 μm. All data are representative of at least three independent experiments. For **a**, a two-tailed unpaired Student's t-test was used; for **f** and **i**, two-way analysis of variance *post hoc* Bonferroni test was used.

Several studies have reported that FoxO family members can induce autophagy that is independent of mTOR signalling^20^,^22^. Given that iNKs undergo mTOR-independent autophagy, we then determined whether FoxO family members initiated autophagy in iNKs. Three FoxO family members, including FoxO1, FoxO3 and FoxO4, are expressed in the hematopoietic system^38^. Interestingly, we found that FoxO1 mRNA was highly transcribed in NKPs, iNKs and mNKs (Fig. 6a,b). However, FoxO3 mRNA level was very low in these NK cells (Fig. 6a,b), and FoxO4 was undetectable. Through immunoblotting, these three FoxO family members were highly expressed in LSKs (Lin^−^Sca1^+^cKit^+^ cells) (Fig. 6b), which were used as a positive control. However, the protein level of FoxO1 was remarkably reduced in mNKs (Fig. 6b). The proteasome inhibitor MG132 was able to block the degradation of FoxO1 and augmented ubiquitinated protein levels in mNKs (Fig. 6c). Similarly, a more specific proteasomal inhibitor epoxomicin also blocked the hydrolysis of FoxO1 in mNKs (Supplementary Fig. 4d). These observations suggest that FoxO1 is degraded in mNKs by the proteasome-dependent degradation pathway. We observed that FoxO1 mainly resided in the nuclei of NKPs (Fig. 6d). However, FoxO1 was mainly distributed in the cytoplasm of iNKs. By contrast, FoxO1 levels were extremely reduced in mNKs (Fig. 6d). These results were further validated by nuclear and cytosolic fractionation assays (Fig. 6e). Furthermore, phosphorylated FoxO1 levels were elevated in the cytoplasm of iNKs (Fig. 6e).

FoxO1 phosphorylation by AKT causes its translocation from the nucleus to the cytosol^39^, leading to transcriptional inactivation of its target genes. FoxO1 activation can upregulate many target genes, including *Il7rα, Bcl2* and *Klf4* (ref. 16), meanwhile, it also downregulates several repressed genes such as *Tbx21, Ccr7* and *Atg7* (refs 17, 18). We observed that expression of genes upregulated by FoxO1 was significantly inhibited during the transition of NKPs to iNKs (Fig. 6f), whereas expression of FoxO1-repressed genes was activated in this process. Additionally, FoxO1 was highly phosphorylated in iNKs (Fig. 6g), while FoxO1 phosphorylation was almost undetectable in mNKs. These observations were validated by immunoblotting (Fig. 6h). As expected, FoxO1 levels were remarkably reduced in mNKs. More importantly, highly phosphorylated FoxO1 was localized in the cytoplasm of iNKs (Fig. 6i), which was confirmed by nuclear and cytosolic fractionation assays (Fig. 6e). In sum, we conclude that phosphorylated FoxO1 resides in the cytoplasm of iNKs (as opposed to the nucleus of NKPs). The differential expression of its target genes in iNKs relative to NKPs is consistent with a loss of FoxO1 transcriptional activity.

### FoxO1 deficiency or mutation abrogates NK cell development

**Figure 7:**
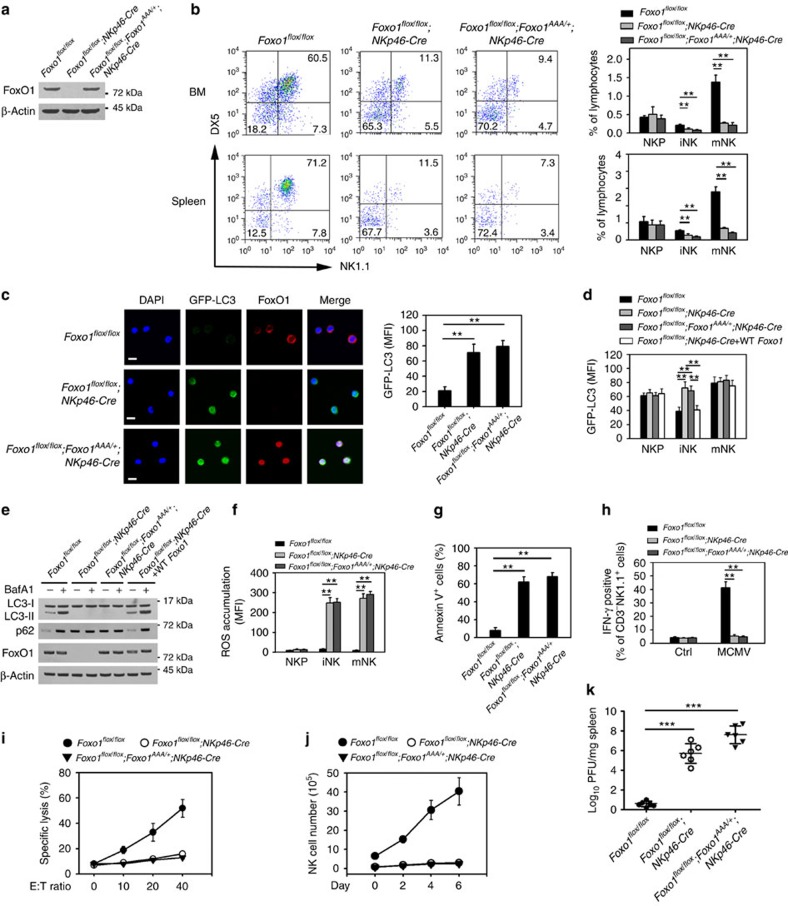
FoxO1 deficiency or Foxo1^AAA^ mutant abolishes autophagy induction and NK cell development. (**a**) FoxO1 of BM NK cells (CD3^−^NK1.1^+^) was deleted in *Foxo*^*flox/flox*^;*NKp46-Cre* mice and *Foxo1*^*flox/flox*^;*Foxo1*^*AAA/+*^*;NKp46-Cre* mice by immunoblotting assay. (**b**) NK cell development is impaired in *Foxo1-deficient* or *Foxo1*^*AAA*^-mutant mice. Propidium iodide (PI)- negative BM and spleen NK cells in *Foxo1*^*flox/flox*^, *Foxo1*^*flox/flox*^*;NKp46-Cre* and *Foxo1*^*flox/flox*^*;Foxo1*^*AAA/+*^*;NKp46-Cre* mice were analysed by flow cytometry. For each group, *n*=7 mice. (**c**) iNK cells were separated from the BM of *GFP-LC3;Foxo1*^*flox/flox*^, *GFP-LC3;Foxo1*^*flox/flox*^*;NKp46-Cre* and *GFP-LC3;Foxo1*^*flox/flox*^*;Foxo1*^*AAA/+*^*;NKp46-Cre* mice for immunostaining. GFP-LC3 MFI of each cell was analysed and show as means±s.d. **P<0.01. Scale bar, 5 μm. (**d**,**e**) For WT FoxO1 rescue, WT NKPs from GFP-LC3 mice were adoptively transferred into *FoxO1*^*flox/flox*^*;NKp46-Cre;GFP-LC3* mice. After three weeks, autophagic flux of NK cells in the indicated mice expressing GFP-LC3 were analysed by flow cytometry (**d**). iNKs from the indicated mice were isolated and treated with BafA1 followed by western blotting (**e**). (**f**) ROS were detected in different NK cells from *Foxo1*^*flox/flox*^, *Foxo1*^*flox/flox*^*;NKp46-Cre* and *Foxo1*^*flox/flox*^*;Foxo1*^*AAA/+*^*;NKp46-Cre* mice. (**g**) Apoptosis of BM CD3^−^NK1.1^+^ NK cells from the indicated mice were stained with Annevin V. (**h**) The indicated mice were infected with MCMV (1 × 10^5^ PFU) for 3 days and intracellular IFN-γ of CD3^−^NK1.1^+^ splenic NK cells were analysed by flow cytometry. (**i**) *Foxo*^*flox/flox*^, *Foxo1*^*flox/flox*^*;NKp46-Cre* and *Foxo1*^*flox/flox*^*;Foxo1*^*AAA/+*^*;NKp46-Cre* mice were infected with MCMV for 3 days. NK cells were separated from spleens and incubated with ^51^Cr-labelled Yac1 cells for cytotoxicity assay. Specific lysis of Yac1 cells was analysed by ^51^Cr release, and shown as means±s.d. (**j**) The indicated mice were infected with MCMV for the indicated days, and NK (CD3^−^NK1.1^+^) cell number was analysed by flow cytometry. (**k**) Virus titres from MCMV-infected *Foxo*^*flox/flox*^*, Foxo1*^*flox/flox*^*;NKp46-Cre* and *Foxo1*^*flox/flox*^*;Foxo1*^*AAA/+*^*;NKp46-Cre* mice were analyzed after 3 days infection (*n*=6 mice). All data represent at least three independent experiments and calculated data are shown as means±s.d. **P<0.01; ***P<0.001. For **b**–**d**,**f**,**g** and **k**, a two-way analysis of variance *post hoc* Bonferroni test was used.

To further confirm the role of FoxO1 in NK cell development, we generated mice with NK cell-specific knockout of Foxo1 gene by crossing *Foxo1*^*flox/flox*^ mice with *NKp46-Cre* mice. FoxO1 was completely deleted in iNKs and mNKs (Fig. 7a). We observed that FoxO1 deficiency impaired NK cell development (Fig. 7b), indicating that FoxO1 plays a critical role in NK cell development. FoxO1 undergoes phosphorylation at three residues (Thr24, Ser256 and Ser319) by AKT^16^. A mutant version of FoxO1, FoxO1^AAA^ (residues Thr24, Ser256 and Ser319 are mutated to Ala) cannot be phosphorylated and remains in the nucleus, leading to its lasting activation of its target genes^39^. FoxO1^AAA^ mice were generated by insertion of FoxO1^AAA^ mutant into Rosa26 locus preceded by a loxP-flanked ‘STOP' cassette^17^. We generated *FoxO1*^*AAA*^-mutant mice (*FoxO1*^*flox/flox*^*;FoxO1*^*AAA/+*^*;NKp46-Cre*) that lacked endogenous expression of WT FoxO1 in NK cells and expressed the *FoxO1*^*AAA*^-mutant gene under the control of *NKp46* promoter^17^. *FoxO1*^*AAA*^ was expressed in the NK cells of these mice (Fig. 7a). Interestingly, the FoxO1^AAA^ mutant also impaired NK cell development (Fig. 7b). The mutant FoxO1 was restricted to nuclei of iNKs (Fig. 7c), while endogenous FoxO1 resided in the cytoplasm of iNKs in *FoxO1*^*flox/flox*^ mice. Consequently, nuclear location of FoxO1^AAA^ mutant abrogated autophagy induction in iNK cells (Fig. 7c–e). Notably, restoration of WT FoxO1 in *FoxO1*^*flox/flox*^*;NKp46-Cre* mice rescued the autophagic activity of iNKs (Fig. 7d,e), suggesting that FoxO1 is essential for autophagy initiation in iNKs.

The suppression of genes usually activated by FoxO1 in iNKs relative to NKPs was not affected in *FoxO1*^*flox/flox*^*;NKp46-Cre* mice compared with *FoxO1*^*flox/flox*^ or *FoxO1*^*flox/flox*^*;NKp46-Cre* rescued with WT FoxO1 (Supplementary Fig. 5a). Likewise the increased expression of genes usually repressed by FoxO1 in iNKs relative to NKPs was unaffected in these genotypes. However, this differential expression of genes usually regulated by FoxO1, which we presume is due to a loss of FoxO1 nuclear localization in iNKs relative to NKPs was not seen in *FoxO1*^*flox/flox*^*;FoxO1*^*AAA/+*^*;NKp46-Cre* mice where FoxO1 is restricted to the nucleus (Supplementary Fig. 5a).

Similarly, *FoxO1* deficiency resulted in the same defective autophagy phenotype in iNK cells as FoxO1^AAA^-mutant mice. Furthermore, FoxO1-deficient or FoxO1^AAA^-mutant NK cells exhibited high accumulation of ROS (Fig. 7f), and elevated dead cells (Fig. 7g). Overall, we conclude that FoxO1 is involved in the induction of autophagy during NK cell development.

To explore the effect of FoxO1 on anti-MCMV response, we used MCMV to infect FoxO1-deficient or FoxO1^AAA^-mutant mice. We observed that *FoxO1*^*−/−*^ or *FoxO1*^*AAA*^ NK cells almost blocked IFN-γ production after MCMV infection (Fig. 7h), whereas *FoxO1*^*flox/flox*^ mice generated large amounts of IFN-γ. Moreover, *FoxO1*^*−/−*^ or *FoxO1*^*AAA*^ NK cells displayed impaired cytotoxicity (Fig. 7i), and significantly reduced numbers in the periphery (Fig. 7j). Finally, FoxO1-deficient or FoxO1^AAA^-mutant mice were more susceptible to MCMV infection (Fig. 7k). These data suggest that FoxO1-mediated autophagy is required for NK cell development and NK cell-mediated innate immunity.

### FoxO1-binding domain mutants fail to induce autophagy

**Figure 8:**
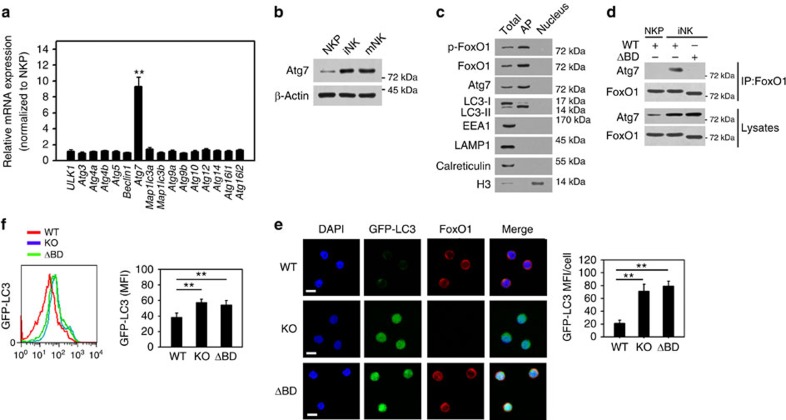
Cytosolic FoxO1 associates with Atg7 for autophagy induction in iNKs. (**a**) mRNA was extracted from NKP and iNK cells followed by RT-PCR. Relative mRNA expression was normalized to that of NKPs. (**b**) NK cells were subjected to Western blotting with the indicated antibodies. (**c**) Autophagosomes of iNKs were isolated and analysed by immunoblotting. LC3-II, autophagosome marker. EEA1, endosome marker. LAMP1, lysosomes marker. Calreticulin, ER marker. H3, nuclear marker. Total, total cell lysates. AP, autophagosomes. (**d**) FoxO1 interacts with Atg7 in iNK cells. NKP or iNK cells from *FoxO1*^*flox/flox*^*;NKp46-Cre* mice cells were infected with lentivirus encoding WT or Atg7 binding domain truncated FoxO1 (ΔBD-FoxO1), and subjected to immunoprecipitation with anti-FoxO1 antibody. (**e**) NKPs were isolated from GFP-LC3 mice and infected with lentivirus encoding WT or ΔBD-FoxO1 or vector, and performed *in vitro* development assay followed by immunostaining of iNK cells. GFP-LC3 MFI of each cell was analysed and shown as means±s.d. **P<0.01. Scale bar, 5 μm. (**f**) Autophagic flux was impaired in ΔBD-FoxO1 NK cells. GFP-LC3 MFI of the indicated iNK cells treated as above was analysed by flow cytometry, and shown as means±s.d. **P<0.01. All data represent at least three independent experiments. For **a**,**e** and **f**, a two-way analysis of variance *post hoc* Bonferroni test was used.

Atg7 is required for formation of autophagosomes^31^. Cytosolic FoxO1 can bind to Atg7 to induce autophagy in human tumour cells independently of its transcriptional activity^20^. We detected autophagy-related genes in iNKs and found that Atg7 was the only autophagy gene differentially regulated in iNKs (Fig. 8a). High expression of Atg7 in iNKs was validated by immunoblotting (Fig. 8b). We next isolated autophagosomes from iNK cells according to a previous study^40^, followed by immunoblotting. Interestingly, we observed that phosphorylated FoxO1 and Atg7 were located in autophagosomes (Fig. 8c), suggesting that phosphorylated FoxO1 and Atg7 could be implicated in the regulation of autophagy in iNKs. Furthermore, the interaction of FoxO1 with Atg7 was verified by immunoprecipitation in iNKs (Fig. 8d). The C-terminal residues 537–596 are the binding domain for Atg7 (ref. 20), deletion of this binding domain of FoxO1 (ΔBD-FoxO1) abrogates its interaction with Atg7. We isolated NKPs and iNKs from the BM of *FoxO1*^*flox/flox*^*;NKp46-Cre* mice and infected with lentivirus encoding WT FoxO1 or ΔBD-FoxO1 for 24 h. With co-immunoprecipitation assays, we noticed that Atg7 associated with FoxO1 in iNK lysates (Fig. 8d). Expectedly, ΔBD-FoxO1 did not interact with Atg7 in iNK lysates (Fig. 8d). We found that ΔBD-FoxO1 ablated autophagy induction in iNKs (Fig. 8e,f), whereas WT FoxO1-induced robust autophagy. Consistently, FoxO1-deficient iNKs did not initiate autophagy (Fig. 8e,f). Moreover, Atg7-silenced iNKs also abrogated autophagy induction and NK cell development (Supplementary Figs 3c and 5b). These data suggest that the interaction of FoxO1 with Atg7 may be essential for the induction of autophagy in iNKs.

**Figure 9:**
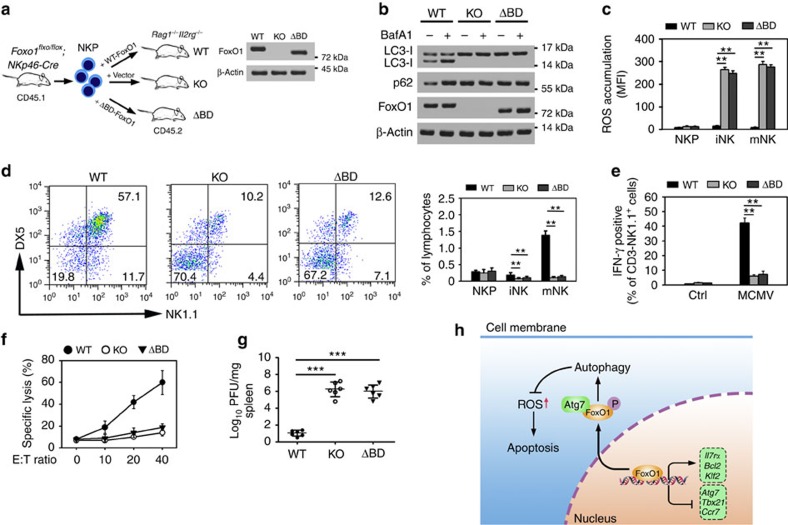
FoxO1-induced autophagy is required for NK cell development and effector functions. (**a**) Scheme for adoptive transfer of NKP cells expressing different constructions of FoxO1 proteins. NKP cells from *Foxo1*^*flox/flox*^*;NKp46-Cre* mice (CD45.1) were infected with lentivirus encoding WT or ΔDB-FoxO1 or vector control, and transferred into *Rag1*^*−/−*^*Il2rg*^*−/−*^ mice (CD45.2). FoxO1 in CD3^+^NK1.1^+^ CD45.1^+^ NK cells was analysed by western blotting. (**b**) Autophagy of iNKs from the indicated mice was analysed by immunoblotting. (**c**) ROS accumulation in NK cells expressing different FoxO1 constructions was analysed by DC-FDA staining. (**d**) After adoptive transfer for 3 weeks, BM CD45.1^+^ NK cells from the indicated mice were analysed by flow cytometry. (**e**) IFN-γ is impaired in ΔBD-FoxO1 NK cells. Intracellular IFN-γ of the CD3^−^NK1.1^+^CD45.1^+^ splenic NK cells from the indicated mice was detected after MCMV infection. (**f**) *Rag1*^*−/−*^*Il2rg*^*−/−*^ mice transferred with WT, knockout or ΔBD-FoxO1 NKPs were infected with MCMV for 3 days. Specific lysis of NK cells were analysed as described in Fig. 7i. (**g**) The indicated mice were infected with MCMV followed by detection of virus titres. *n*=6 mice for each group. All data represent at least three separate experiments and calculated data are shown as means±s.d. **P<0.01; ***P<0.001. (**h**) Work model for FoxO1-induced autophagy during NK cell development. All data represent at least three independent experiments. For **c**,**d**,**e** and **g**, a two-way analysis of variance *post hoc* Bonferroni test was used.

To further confirm the *in vivo* role of FoxO1 in NK cell development and effector functions, we transplanted NKPs expressing WT FoxO1 or ΔBD-FoxO1 into *Rag1*^*−/−*^*Il2rg*^*−/−*^ mice (Fig. 9a). Expression levels of FoxO1 and ΔBD-FoxO1 in transplanted NK cells were comparable (Fig. 9a). Moreover, NKPs isolated from these three mice strains similarly distributed in recipient mouse spleens (Supplementary Fig. 5c). Of note, ΔBD-FoxO1-transplanted NKPs failed to induce autophagy in iNKs (Fig. 9b), and consequently generated a high level of ROS (Fig. 9c). We noticed that FoxO1-deficient iNKs gave similar results to ΔBD-FoxO1-transplanted iNKs. Consequently, ΔBD-FoxO1-transplanted NKPs impaired NK cell development (Fig. 9d). Finally, ΔBD-FoxO1-transplanted NKs impaired their ability to produce IFN-γ (Fig. 9e), and defensive response to MCMV infection (Fig. 9f,g), which was similar to FoxO1 deficiency. Altogether, we conclude that the association of cytosolic FoxO1 with Atg7 likely contributes to the autophagy induction of iNKs that initiates NK cell development and effector functions.

## Discussion

NK cells play a critical role in early immune responses against transformed tumours and virus-infected cells^1^. NK cells develop from BM-derived HSCs and undergo several developmental stages to give rise to mature lineages^8^. However, the underlying mechanisms of NK cell development remain largely elusive. In this study, we show that autophagy is required for NK cell development. FoxO1 and Atg7 are highly expressed in the cytoplasm of iNKs in BM, and when their association is disrupted robust autophagy fails to be initiated (Fig. 9h). Autophagy defects cause ROS accumulation that leads to apoptosis of NK cells. Autophagy is responsible for removal of intracellular ROS, which protects NK cell viability during NK cell specification. During NK cell development, NK cells express activating receptors and inhibitory receptors, as well as synthesize cytotoxic granules, which make NK cells ready for immune responses^7^. We also demonstrate that FoxO1-mediated autophagy is essential for effector functions of NK cells against viral infection.

Autophagy harbours multiple effects on immunity^23^. Invading microorganisms induce autophagy by competing nutrients or by activating innate immune receptors such as Toll-like receptors (TLRs). Autophagosomes engulf microbes and degrade them by recognition of autophagic adaptors, known as sequestosome 1-like receptors^41^. Recently, autophagy has been considered to be implicated in innate and adaptive immune responses^24^. Lack of autophagy affects lymphoid precursor formation^42^. Autophagy deficiency abolishes both negative and positive selection of CD4^+^ T cells^43^. Naive T cells require autophagy for their maturation after exiting the thymus^44^. Autophagy is also needed for B1 cell survival^45^. Additionally, plasma cells also require autophagy to sustain their viability during differentiation and to maintain their long-lived humoral immunity^26^. For the first time, we demonstrate that autophagy is essential for NK cell development and effector responses against viral infection.

ROS refer to O_2_-free radicals and non-free radicals' derivatives. ROS are mainly produced from mitochondria accompanying mitochondrial respiration. Moderate ROS levels are required for hematopoiesis during embryonic development and adult hematopoietic homoeostasis^46^. However, high levels of ROS can cause DNA damage, mitochondrial dysfunction and harmful protein modifications^47^. Since mitochondria are both the producers and targets of ROS, dysfunctional mitochondria can generate excessive ROS and release apoptosis-related factors such as cytochrome c and AIF (apoptosis inducing factor), which result in cell death. Increased ROS content triggers stem cell differentiation to short-term repopulating cells^48^. Here we show that ROS are kept low levels in NKPs, iNKs and mNKs in wild-type (WT) mice. Autophagy deficiency causes damaged mitochondria and substantial ROS accumulation in iNKs and mNKs, which lead to apoptosis of NK cells and blockade of NK cell maturation. Autophagy scavengers are able to counteract this effect, suggesting that autophagy is responsible for removal of ROS during NK cell development. Additionally, high ROS levels can inhibit effector functions of NK cells^49^. Thus, removal of high ROS accumulation could also be needed for the immune responses of NK cells.

Blockade of mTOR signalling is the most well-known trigger for autophagy initiation. Inhibition of mTOR leads to the activation of ULK1 complex that initiates autophagy^50^. We found mTOR is activated during NK cell development. Blockade of mTOR by rapamycin impairs, but does not promote, autophagy during NK cell maturation. Additionally, depletion of Ractor or Rictor in iNKs suppresses rather than promotes autophagy induction. Thus we conclude that autophagy induction in iNKs is independent of mTOR signalling. A recent study showed that a high dose of IL-15 induces mTOR activation and elevated bioenergetic metabolism in NK cells^51^. mTOR is an essential component of IL-15-mediated signalling, which is crucial for NK cell commitment. We observed that blocking IL-15 signalling abrogates autophagy induction in iNKs (Supplementary Fig. 5d), suggesting IL-15 signalling might be a upstream stimulus for autophagy initiation. mTOR activation is needed for the signalling transduction in PI3K-AKT pathway^35^,^52^. Transduction of IL-15 signalling relies on the PI3K-AKT pathway^53^. Actually, we show that the PI3K-AKT pathway is activated during NK cell maturation. Therefore, the active mTOR signalling is needed to activate the PI3K-AKT pathway for NK cell lineage specification.

FoxO transcription factors take part in many physiological processes, including integration of growth factor signalling, oxidative stress and immune responses^16^. FoxO1, a FoxO family member, regulates cell cycle, cell metabolism and apoptosis as a transcription factor^39^. FoxO1 is crucial for the fate determination of T cells^16^. Activation of FoxO1 drives T cells towards Treg cells or CD8^+^ memory T cells^17^. Here we found that FoxO1 resides in the autophagosomes of iNKs and its interaction with Atg7 initiates autophagy induction. We noticed that blocking IL-15 signalling abolishes phosphorylation of FoxO1 and consequent translocation to the cytoplasm of iNKs (Supplementary Fig. 5e), leading to autophagy inhibition. By contrast, cytosolic FoxO1 is rapidly hydrolysed in the cytoplasm of mNKs, which impairs autophagy induction. We still need to further investigate how cytosolic FoxO1 is degraded in mNKs. Thereby, the autophagic activity of FoxO1 is essential for NK cell maturation, which is independent of its transcriptional activation of its target genes.

A recent study reported a transcription-independent role of FoxO1 (ref. 20). Cytosolic FoxO1 dissociates from its deacetylase SIRT2 and induces its acetylation. Acetylated FoxO1 interacts with Atg7 to initiate autophagy in tumour cells^20^, which exerts antitumour activity. Here we show that phosphorylated cytosolic FoxO1 associates with Atg7 to induce autophagy in iNKs, which is essential for NK cell development. Whether the acetylation of FoxO1 is also needed for interaction with Atg7 in iNKs remains to be further explored. Cytosolic FoxO1 is dramatically degraded in mNKs, which may impede the interaction of FoxO1 with Atg7 leading to inhibition of autophagy. We propose that the modifications of FoxO1 may promote its interaction with Atg7, which stabilizes FoxO1 in the cytoplasm of iNKs resulting in autophagy induction. The subcellular localization of FoxO1 depends on post-translational modifications, especially phosphorylation^54^. Localization of FoxO1 depends on a variety of kinases such as AKT^54^. AKT is highly active in iNKs, suggesting that AKT-mediated phosphorylation of FoxO1 could contribute to the translocation of FoxO1 to the cytosol of iNK cells. We found that FoxO1 is uniquely localized in the nuclei of NKPs, and it resides in the cytoplasm of mNKs and iNKs.

Recently, Deng *et al*.^55^ reported that FoxO1 is dispensable for NK cell development, and inactivation of FoxO1 is required for T-bet expression. The reasons for the apparent discrepancy between the results of Deng *et al*. and the current study are currently unclear. NKp46-Cre mice were generated independently in this study but we did follow the same procedure as described in Deng *et al*.^55^. In our study, as in Deng *et al*.^55^ we found that the transcriptional activity of FoxO1 is impaired in iNKs (Supplementary Fig. 5a). However, we failed to detect FoxO1 signals in mature NKs by using an anti-FoxO1 antibody (D7C1H, CST). Consistent results were obtained using two other anti-FoxO1 antibodies from another company (C-9 and H-128, Santa Cruz). In addition, we found FoxO1 underwent phosphorylation and proteasome-dependent degradation in mNKs (Fig. 6c). Deng *et al*.^55^ also found FoxO1 was phosphorylated during NK cell maturation. By contrast, Deng *et al*. used a different batch of anti-FoxO1 antibody (C29H4, CST) to detect FoxO1 signals by flow cytometry. Possible reasons for detecting high FoxO1 levels in mNKs by Deng *et al*. may be as follows: (1) the C29H4 antibody against FoxO1 could harbour stronger affinity to probe FoxO1 in mNK cells; (2) for flow cytometry assays, staining buffers could impair proteasome function in mNKs abrogating the degradation of FoxO1. In our experimental assays, we used propidium iodide staining to exclude necrosis and late stage apoptotic cells. Additionally, we stained cells with Annexin V to analyse early apoptotic cells for flow cytometry assays. However, Deng *et al*. did not state the exclusion of dead cells in their assay systems^55^. Flow cytometry was the major assay for cell number calculations in their experiments. Dead cells could potentially impact the number counts of NK cells by flow cytometry analyses. Another possible explanation for this difference is that autophagy induction is a very dynamic response susceptible to different environments and body metabolism^56^,^57^. Different food, water, living environments and metabolic states of mice might cause different regulations for autophagy induction. We hypothesize that the mice with different feeding conditions might cause distinct autophagic regulations that affect NK cell development. Finally, subtle expression differences between NKp46-Cre lines could be another potential explanation for the differences.

In conclusion, we reveal that FoxO1-mediated autophagy in iNKs is required for NK cell development and NK cell-mediated innate immunity, which may provide a novel strategy to promote NK cell activity by manipulating FoxO1 for future clinical applications.

## Methods

### Antibodies and reagents

Antibodies used for flow cytometry: anti-NK1.1 (PK136,1:200), anti-CD122 (5H4, 1:200), anti-NKp46 (29A1.4, 1:100), anti-DX5 (DX5, 1:200), anti-CD94 (18D3, 1:100), anti-CD19 (1D3, 1:300), anti-CD11b (M1/70, 1:300), anti-CD117 (2B8, 1:100), anti-IL-7Rα (A7R34, 1:200), anti-Gr-1(RB6-8C5, 1:300), anti-CD45.1 (A20, 1:500), CD45.2 (104, 1:500), and anti-mouse hematopoietic lineage cocktail (CD3(17A2, 1:300), CD19 (1D3, 1:300), TER-119 (TER-119, 1:200), Gr-1 (RB6-8C5, 1:300)) were purchased from eBiosicence. Antibodies against FoxO1(#14952, 1:500), p-FoxO1 (Ser319,#9461, 1:300), FoxO3 (#2497, 1:500), FoxO4(#9472, 1:500), p-mTOR(Ser 2448, #5536, 1:500), p-AKT (Ser473, #5012, 1:500), p-AKT(Thr308, #13038, 1:500), p-AMPKα1 (Thr172, #5256, 1:500), p-AMPKβ1 (Ser108, #4181, 1:500), p-TSC2 (Tyr1462, #3617, 1:500), p-4EBP (Thr37/Thr46, #2846, 1:300), p-S6 Kinase (Thr389, #9206, 1;300), Atg7(#8558, 1:500), Atg5 (#12994, 1:500), Atg3 (#3415, 1:500), activated caspase3 (#9579, 1:500), EEA1 (#3288, 1:500)and Histone H3 (#4499, 1:500) were from Cell Signaling Technology. Anti-LC3 (#PM036, 1:1000)and anti-p62 (#PM045, 1:1,000) antibodies were from MBL. Propidium iodide, rapamycin and DCF-DA were from Sigma-Aldrich.

### Mice

*GFP-LC3* mice^58^ and *Atg5*^*flox/flox*^ mice^59^ were from the Riken Bio Resource Center (Japan). *NKp46-Cre* mice were generated by Beijing Biocytogen (Beijing, China). *FoxO1*^*flox/flox*^ and *FoxO1*^*AAA/+*^ mice are kind gifts from Dr Ming Li^17^(Memorial Sloan-Kettering Cancer Center, USA). Mice with NK cell-specific deletion of Atg5 were generated by crossing *Atg5*^*flox/flox*^ mice with NKp46-Cre mice. Mice with NK cell-specific expression of FoxO1 were generated by crossing *FoxO1*^*flox/flox*^*;FoxO1*^*AAA/+*^ mice with NKp46-Cre mice. *Rag1*^*−/−*^ mice were from Model Animal Research Center of Nanjing University. *Il2rg*^*−/−*^ mice were from Jackson Laboratories. *Rag1*^*−/−*^*Il2rg*^*−/−*^ mice were generated by crossing *Rag1*^*−/−*^ mice with *Il2rg*^*−/−*^ mice. Both female and male mice were used in our experiments. All the mice are C57BL/6 background with age of 8–12 weeks and maintained under specific pathogen-free conditions with approval by the institutional committee of Institute of Biophysics, Chinese Academy of Sciences.

### Isolation of NK populations

Total bone marrow cells were flushed from mouse femurs and stained with antibodies against lineage, CD122, NK1.1 and DX5. Different stages of NK cells were isolated by flow cytometry. Purity of each NK population was examined by flow cytometry. NKP, Lin^−^CD122^+^NK1.1^−^DX5^−^; iNK, Lin^−^CD122^+^NK1.1^+^DX5^−^; mNK, Lin^−^CD122^+^NK1.1^+^DX5^+^. Lineage cocktails (Lin) included CD3 (17A2), CD19 (1D3), TER-119 (TER-119) and Gr-1 (RB6-8C5).

### Purification of autophagosomes

Autophagosomes from NK cells were isolated according to a technical report^40^. NK cells (1 × 10^6^) were isolated by flow cytometry, resuspended by ice-cold 10% (w/v) unbuffered, electrolyte-free sucrose and centrifuged for 5 min at 1,400 r.p.m. Pellets were homogenized in homogenization buffer (10 mM HEPES, 1 mM EDTA, pH 7.3). Homogenates were treated with homogenization buffer 1.5 mM glycyl-L-phenylalanine 2-naphthylamide and 1% DMSO to remove lysosomes, with Nycodenz solution to remove mitochondria, with 33% solution of Percoll to remove small-vesicular and membraneous materials.

### Adoptive cell transfer experiment

NKP cells (CD122^+^NK1.1^−^Lin^−^) (5 × 10^5^) were separated from BM of *FoxO1*^*flox/flox*^*;NKp46-Cre* mice (CD45.1), and infected with lentivirus encoding WT or ΔBD-FoxO1 protein. Next, NKPs expressing WT or ΔBD-FoxO1 protein were transferred into *Rag1*^*−/−*^*Il2rg*^*−/−*^ mice (CD45.2) for NK cell reconstitution. After 3 weeks, mice were killed for flow cytometry analysis or MCMV infection.

### Immunofluorescence assay

For *in situ* immunofluorescence of NK cells, femurs from the indicated mice were fixed in paraformaldehyde-lysine-periodate fixative for 8 h, and decalcified in decalcifying buffer (10% EDTA in PBS (w/v), pH 7.4). Then femurs were rehydrated in 30% sucrose solution for 24 h and frozen in OCT for sectioning. BM sections were rehydrated in PBS and blocked in 10% donkey serum. Next, the sections were incubated with primary antibodies (anti-NKp46 (R&D), DX5 (eBioscience) and anti-B220 (BD), and anti-activated caspase3 (Cell Signaling)) for 2 h at room temperature followed by washing in 0.1% Tween20/PBS for three times. Sections were then incubated with Alexa488-, Alexa594- or Alexa405-conjugated secondary antibodies (Invitrogen). After washing for three times, DAPI was added and subjected to dehydrating in EtOH gradient: 70, 85, 95, 100%, 3 min each. Mounted sections were analysed by confocal microscopy (Olympus FV1000). For cell immunofluorescence, NK cells at different stages were isolated by flow cytometry, and fixed on 0.01% poly-L-Lysine treated coverslips with paraformaldehyde (Sigma-Aldrich) for 20 min at RT. Then cells were permeabilized, and stained with primary and secondary antibodies.

### *In vitro* NK cell development assay

NKP cells were isolated from BM of the indicated mice, and infected with lentivirus encoding different shRNA constructions. NKPs were then cultured on OP9 stroma cells with RPMI1640 medium containing 10% fetal bovine serum (FBS), 100 μg ml^−1^ streptomycin and 100 U ml^−1^ penicillin, and 20 ng ml^−1^ of IL-15 for 7 d followed by flow cytometry.

### *In vitro* cytotoxicity assay

NK cells from mouse spleen were isolated. Yac1 cells (from ATCC) were labelled with 200 μCi ^51^Cr at 37 °C for 1 h and plated at 1 × 10^4^ cells per well. NK cells were incubated with Yac1 cells at the indicated E/T ratios for 4 h, and the supernatants were harvested for the detection of specific ^51^Cr release using MicroBeta counter (PerkinElmer). Specific cytotoxicity was calculated as [(sample release-spontaneous release)/(maximum release-spontaneous release)] × 100. Maximum release of Yac1 cells was measured by treatment with 2% TritonX-100.

### Electron microscopy

For immuno-EM, 1 × 10^6^ NK cells at different stages form WT mice were isolated. and fixed with 4% paraformaldehyde and 0.05% glutaraldehyde for 2 h on ice and then cryo-sectioned by Tokuyasu method and mounted in copper grids followed by blocking in 1% BSA. Sections were stained with anti-LC3 (30 ng μl^−1^) antibody (PM036, MBL) and further stained with 10 nM gold linked anti-rabbit IgG. Then the sections were fixed with 2.5% glutaraldehyde and stained with neutral uranyl acetate before coating with 2% methyl cellulose. Images were taken with a FEI Tecnai spirit transmission electron microscope.

### RNA interference

shRNAs against Atg3, Atg7, Raptor or Rictor were constructed into pSIN-EF2 vector. Lentiviruses encoding RNA interference sequences were generated by transfecting pSIN-EF2-shRNA and packaging vectors into HEK293T cells, followed by ultracentrifugation concentration. The RNA interference sequences were previously described^25^.

### Real-time PCR assay

NK cells were isolated from the indicated mice, mRNA was extracted by using Neasy Micro Kit (Qiagen) according to the manufacturer's instructions. After extraction of mRNA, quality of mRNA was determined by A260/A280 (between 1.8 and 2.2) and A260/230 (>1.7) ratios. RNA integrity was assessed by agarose gel. Two major bands corresponding to the 28S and 18S rRNA were clearly visible. Next, cDNAs were synthesized by using oligo dT followed by real-time PCR. Efficiency of each pair primer was above 90%. Primers for real-time PCR were used as described before^25^. Other primers for real-time PCR in this study are as follows: FoxO1-F: 5′-CCCAGGCCGGAGTTTAACC-3′, FoxO1-R: 5′-GTTGCTCATAAAGTCGGTGCT-3′; FoxO3-F: 5′-CTGGGGGAACCTGTCCTATG-3′, FoxO3-R: 5′-TCATTCTGAACGCGCATGAAG-3′; FoxO4-F: 5′-CTTCCTCGACCAGACCTCG-3′, FoxO4-R: 5′-ACAGGATCGGTTCGGAGTGT-3′; Tbx21-F: 5′-AGCAAGGACGGCGAATGTT-3′, Tbx21-R: 5′-GGGTGGACATATAAGCGGTTC-3′; Il-7rα-F: 5′-GACTACAGAGATGGTGACAG-3′, Il-7rα-R: 5′-GGTGACATACGCTTCTTCT-3′; Klf2-F: CTCAGCGAGCCTATCTTG-3′, Klf2-R: 5′-AGAGGATGAAGTCCAACAC-3′. Relative quantification of mRNA was determined by the ΔΔCt method.

### MCMV infection

Mice were intraperitoneally injected with 1 × 10^5^ PFU MCMV (VR-1399, Smith strain from ATCC). After 72 h, mice were sacrificed for the collection of spleens. Intracellular IFN-γ in NK cells was analysed by flow cytometry. For determination of virus titres, the spleens from infected mice were homogenated in 2 ml of complete cold medium followed by standard plaque-forming assay.

### Statistical analysis

For statistical analysis, two-tailed unpaired Student's t-test and two-way analysis of variance *post hoc* Bonferroni test were used with Sigma Plot and GraphPad softwares. We calculated medians of fluorescence intensity by using FlowJo software for flow cytometry data, and using Image J software for confocal data. We calculated s.d. by using SigmaPlot for at least four independent experiments.

The uncropped data for blot and gel images can be found in Supplementary Figs 6–9.

## Additional information

**How to cite this article:** Wang, S. *et al*. FoxO1-mediated autophagy is required for NK cell development and innate immunity. *Nat. Commun.* 7:11023 doi: 10.1038/ncomms11023 (2016).

## Supplementary Material

Supplementary Information Supplementary Figures 1-11
