# Supplementary material for: FoxO1-mediated autophagy is required for NK cell development and innate immunity
Source: Nat Commun. 2016 Mar 24;7:11023. doi: 10.1038/ncomms11023 (PMC4820827; doi:10.1038/ncomms11023)
Supplement: Supplementary Information — Supplementary Figures 1-11 [file ncomms11023-s1.pdf]

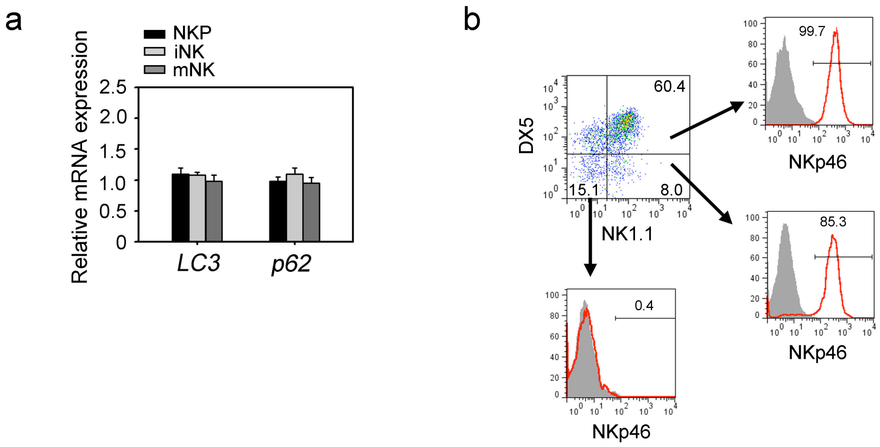

Supplementary Figure 1. Autophagy is required for NK cell development. (a) mRNA levels of LC3 and p62 in NK cells. NK cells were separated from bone marrow (BM) of WT mice and lysed for mRNA extraction. Relative mRNA levels were analyzed by RT-PCR. (b) NK cells from WT BM were analyzed by flow cytometry for expression of NK1.1 and NKp46. All data represent at least three independent experiments and calculated data are shown as means±SD. For a, two-tailed unpaired Student's t-test was used.

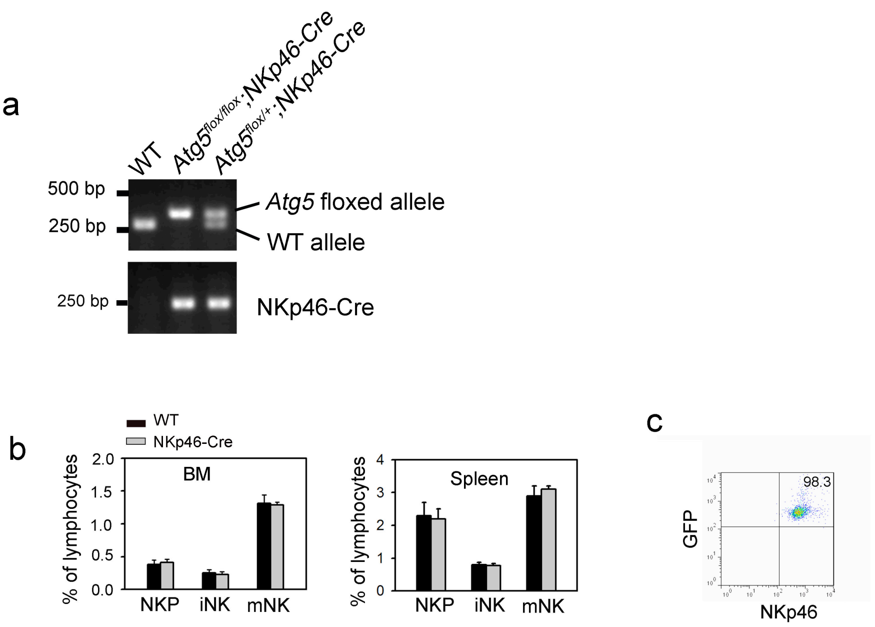

Supplementary Figure 2. Identification of *Atg5$^{flox/+}$;NKp46-Cre* and *Atg5$^{flox/flox}$;NKp46-Cre* mice. (a) Genotyping of *Atg5$^{flox/+}$;NKp46-Cre* and *Atg5$^{flox/flox}$;NKp46-Cre* mice. Genomic DNA of the indicated mice was extracted for PCR assay. (b) NK cell development in WT and *NKp46-Cre* mice. NK cells at different stages from BM or spleen of WT and *NKp46-Cre* mice were analyzed by flow cytometry. (c) GFP and NKp46 were analyzed in *NKp46-Cre;Rosa-flox-STOP-flox-GFP* mice. All data represent at least three independent experiments and calculated data are shown as means±SD. For b, two-tailed unpaired Student's t-test was used.

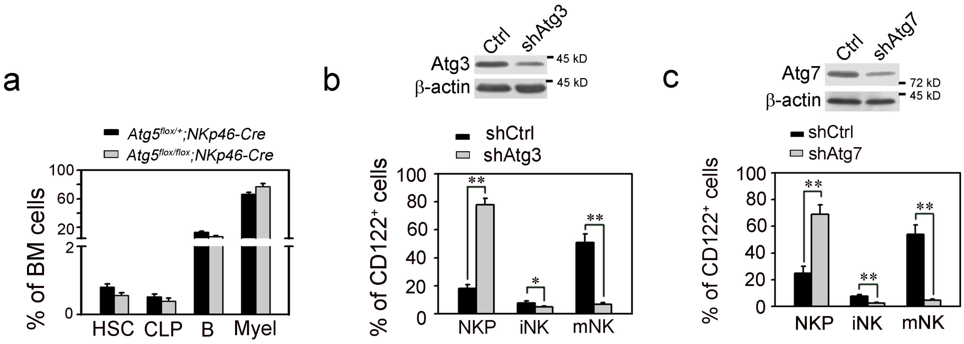

Supplementary Figure 3. Autophagy is required for NK cell maturation. (a) Deletion of *Atg5* in NK cells does not affect the numbers of other lineage cells in BM. Percentages of the indicated lineage cells were analyzed by flow cytometry. Myel, myeloid cell. (b) Atg3 silencing impairs NK cell development. NKP cells were isolated from WT BM and infected with lentivirus encoding shAtg3. 7 d after cultured with IL-15, cells were examined for Atg3 expression by Western blotting and analyzed by flow cytometry. (c) Atg7 is essential for NK cell development. Atg7 silenced NKP cells were subjected to in vitro development assay and analyzed as above. All data represent at least three independent experiments and calculated data are shown as means±SD. $*$, $P<0.05$; $**$, $P<0.01$. For a-c, two-tailed unpaired Student's t-test was used.

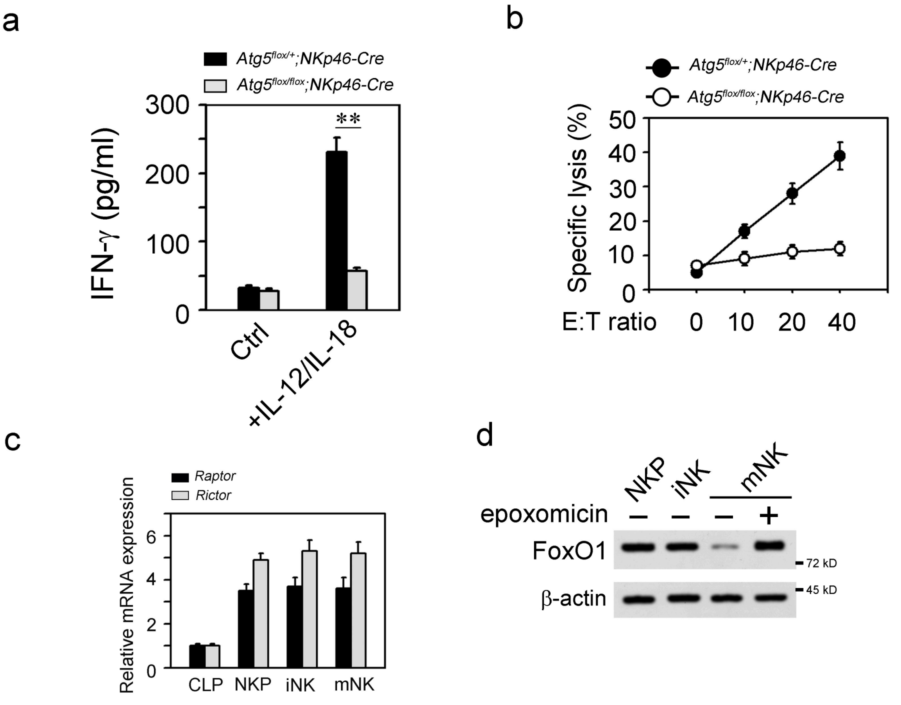

Supplementary Figure 4. Autophagy is required for NK cell effector functions. (a) Mature NKs were isolated from the indicated mice and treated with 10 ng/ml IL-12 and IL-18 for 12 h. Secreted IFN-γ was analyzed by ELISA. (b) Isolated mNKs from the indicated mice were treated with 10 ng/ml IL-2 for 4 days and incubated with ^{51}Cr labeled Yac1 cells with different E:T ratios. Specific lysis of Yac1 was calculated by ^{51}Cr release, and shown as means±SD. (c) Expression of Raptor and Rictor was analyzed by RT-PCR and shown as means±SD. (d) Different NK cell populations were isolated and mNKs were treated with 10 μM epoxomicin for 6 h followed by immunoblotting. All data represent at least three independent experiments and calculated data are shown as means±SD. **, *P*<0.01. For a-c, two-tailed unpaired Student's t-test was used.

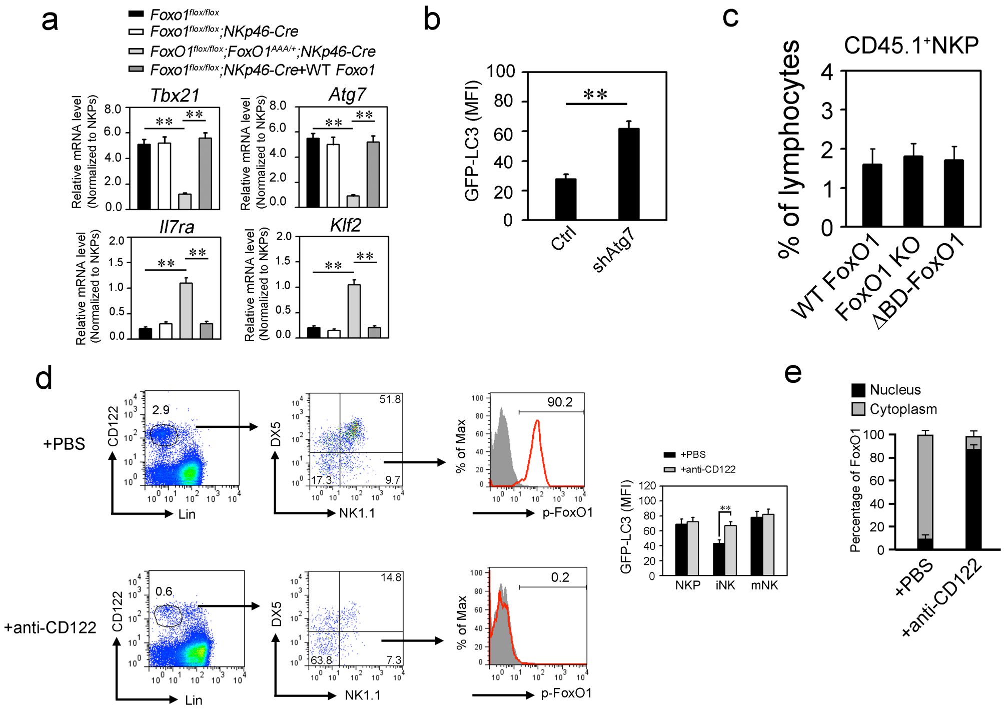

Supplementary Figure 5. Cytosolic FoxO1 is required for NK cell maturation. (a) Analysis of FoxO1 target genes by RT-PCR in iNKs from *FoxO1$^{flox/flox}$*, *FoxO1$^{flox/flox}$;NKp46-Cre*, *FoxO1$^{flox/flox}$;FoxO1$^{AAA/+}$;NKp46-Cre* and WT FoxO1-rescued (*FoxO1$^{flox/flox}$;NKp46-Cre* + WT *Foxo1*) mouse strains. Relative mRNA expression was normalized to that of NKPs. (b) Atg7 silencing impairs autophagic activity in iNKs. (c) NKPs from the indicated mice were isolated and transferred into *Rag1$^{-/-}$Il2rg$^{-/-}$* mice for 2 weeks. NKPs from recipient mouse spleen were analyzed by flow cytometry. (d) GFP-LC3 mice were injected *i.p.* with PBS or 200 μg of anti-CD122 antibody (TM-β1) in 0.2 ml of sterile PBS for 2 weeks. NK cells from BM were analyzed by flow cytometry. Medians of GFP fluorescence intensity (MFI) of each cell were calculated and shown as means±SD. ∗∗, *P*<0.01. (e) Percentages of nuclear and cytoplasmic FoxO1 in isolated iNKs from the indicated mice were analyzed and shown as means±SD. Data represent at least four independent experiments and calculated data are shown as means±SD. ∗∗, *P*<0.01. For a-e, two-tailed unpaired Student's t-test was used.

Fig.1d anti-LC3

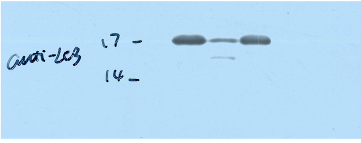

Fig.1d anti-LC3

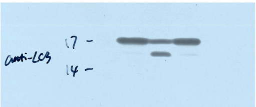

Fig.1d anti-p62

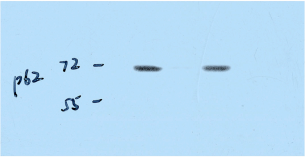

Fig.1d anti-p62

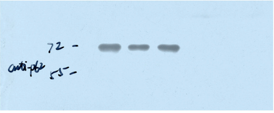

Fig.1d anti-β-actin

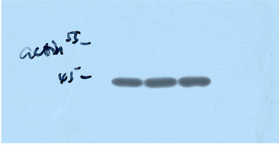

Fig.1d anti-β-actin

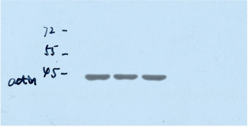

Fig.2a, anti-Atg5

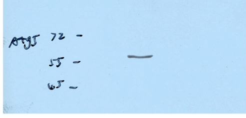

Fig.2a, anti-β-actin

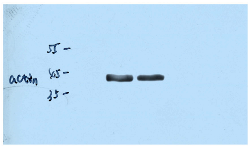

Supplementary Figure 6. Uncropped data for Figure 1-2.

Fig.5c, anti-Raptor

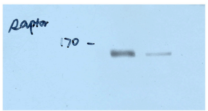

Fig.5e, anti-Rictor

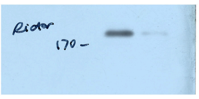

Fig.5c, anti-β-actin

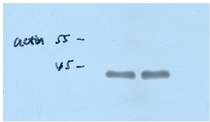

Fig.5e, anti-β-actin

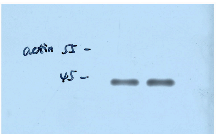

Supplementary Figure 7. Uncropped data for Figure 5.

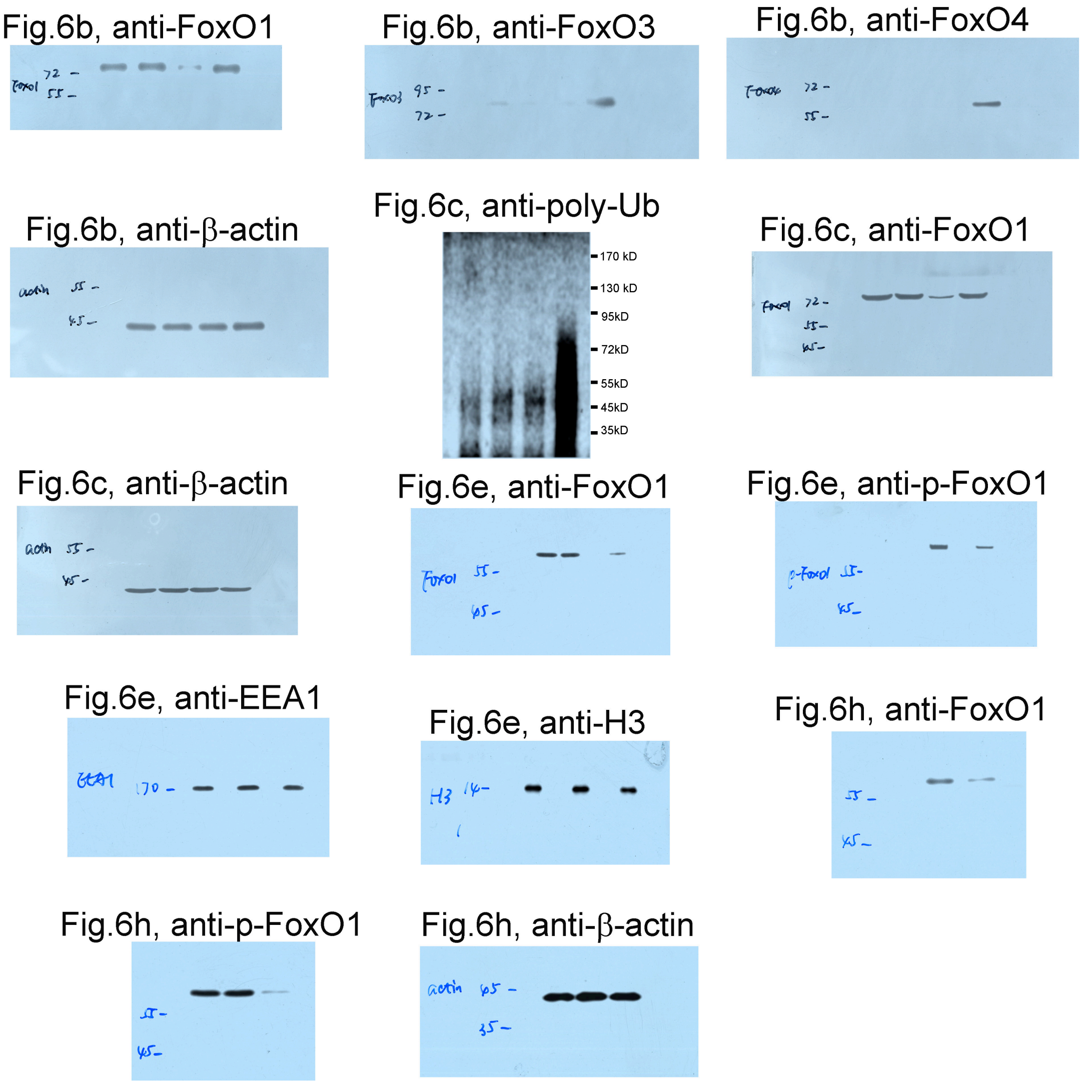

Supplementary Figure 8. Uncropped data for Figure 6.

Fig.7a, anti-FoxO1

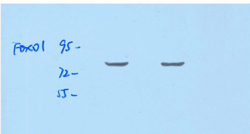

Fig.7a, anti-β-actin

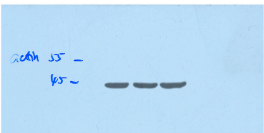

Fig.7e, anti-LC3

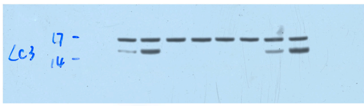

Fig.7e, anti-p62

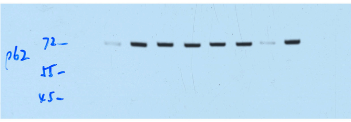

Fig.7e, anti-FoxO1

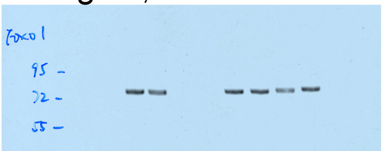

Fig.7e, anti-β-actin

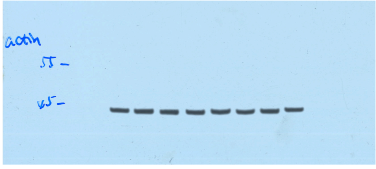

Supplementary Figure 9. Uncropped data for Figure 7.

Fig.8b, anti-Atg7

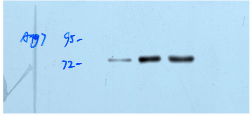

Fig.8b, anti-actin

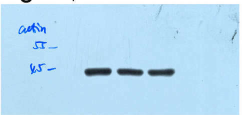

Fig.8d, anti-Atg7

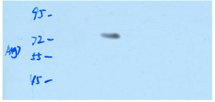

Fig.8d, anti-FoxO1

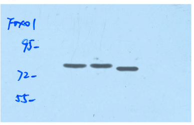

Fig.8d, anti-Atg7

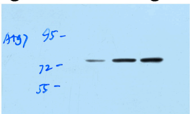

Fig.8d, anti-FoxO1

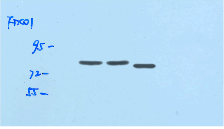

Fig.8c, anti-p-FoxO1

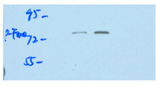

Fig.8c, anti-FoxO1

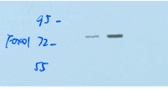

Fig.8c, anti-Atg7

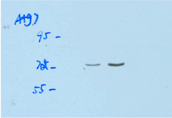

Fig.8c, anti-LC3

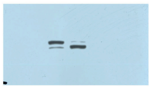

Fig.8c, anti-EEA1

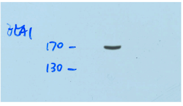

Fig.8c, anti-LAMP1

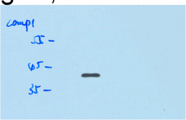

Fig.8c, anti-calreticulin

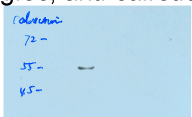

Fig.8c, anti-H3

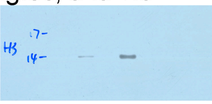

Fig.9a, anti-FoxO1

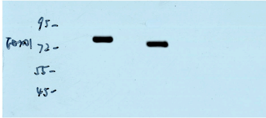

Fig.9a, anti-actin

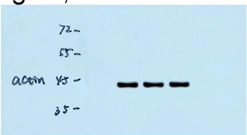

Fig.9b, anti-LC3

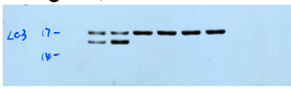

Fig.9b, anti-p62

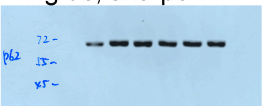

Fig.9b, anti-FoxO1

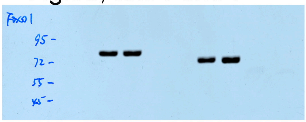

Fig.9b, anti-actin

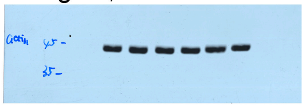

Supplementary Figure 10. Uncropped data for Figure 8-9.

Fig.S2a Atg5^{flox} primer

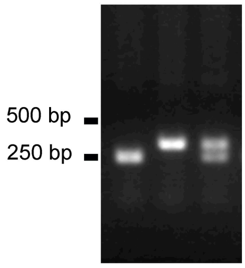

Fig.S2a NKp46-Cre primer

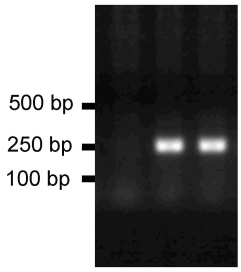

Fig.S3b, anti-Atg3

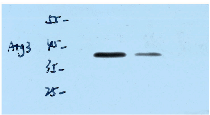

Fig.S3c, anti-Atg7

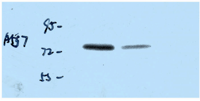

Fig.S3b, anti-β-actin

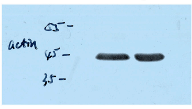

Fig.S3c, anti-β-actin

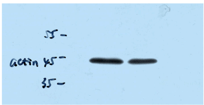

Fig.S4d, anti-FoxO1

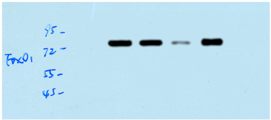

Fig.S4d, anti-β-actin

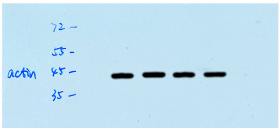

Supplementary Figure 11. Uncropped data for Supplementary Figure 2-4.
